# Microsomal triglyceride transfer protein restricts steroid production in Leydig cells by regulating SREBP2

**DOI:** 10.1016/j.isci.2026.115817

**Published:** 2026-04-20

**Authors:** Atrayee Chattopadhyay, Munichandra Babu Tirumalasetty, Thomas Palaia, Bhargavi Gangula, Rachel Ruoff, Qing Robert Miao, M. Mahmood Hussain

**Affiliations:** 1 Department of Foundations of Medicine, NYU Grossman Long Island School of Medicine, Mineola, NY, USA; 2 Department of Biochemistry and Molecular Pharmacology, NYU Grossman School of Medicine, Manhattan, NY, USA

**Keywords:** Molecular biology, Endocrinology

## Abstract

Testicular Leydig cells produce testosterone. Decreases in serum testosterone reduce bone density, muscle mass, and sexual functions, and increase obesity and cardiovascular disease. Microsomal triglyceride transfer protein (MTP) is critical for lipoprotein secretion by the liver and intestine. MTP is expressed in the Leydig cells of human and mouse testes, but its function is unknown. MTP inhibition, knockdown, and gene ablation in mouse Leydig cells significantly increased steroid secretion after stimulation with cyclic AMP. MTP deficiency enhanced the expression of cholesterol metabolism genes, luteinizing hormone receptor, and steroid biosynthesis genes. Furthermore, MTP deficiency enhanced the expression and activity of SREBP2. ATAC sequencing revealed enhanced binding of SREBP2 to the promoters of genes in cholesterol metabolism and steroid biosynthesis pathways. SREBP2 knockdown in MTP-deficient cells significantly attenuated the cholesterol and progesterone biosynthesis pathways. These studies show that MTP regulates cholesterol and sex hormone production by modulating SREBP2 activity.

## Highlights

• MTP deficiency in Leydig cells increased steroidogenesis and cholesterol metabolism
• MTP-deficient MA-10 cells showed elevated SREBP2 activity
• *Srebf2* knockdown in MTP-deficient cells significantly attenuated steroid biosynthesis
• MTP deficiency enhanced SREBP2 binding at the cholesterol and steroidogenic gene promoters

## Graphical abstract

**Figure:**
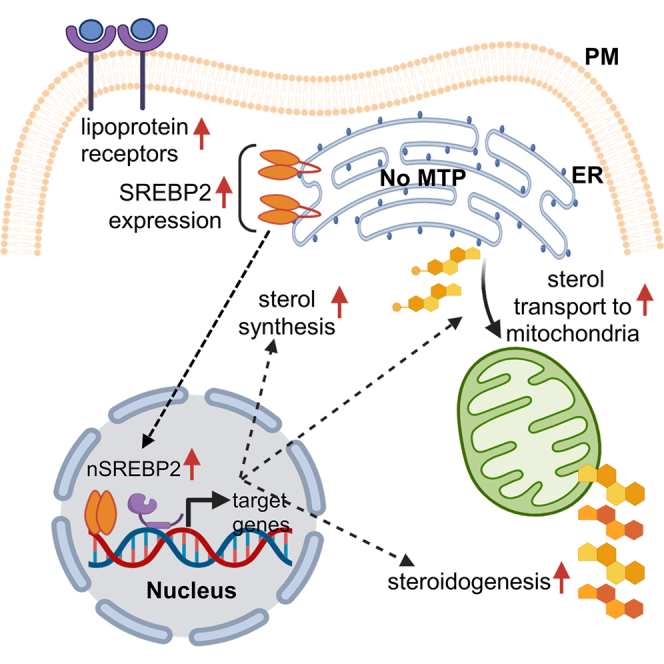

## Introduction

Microsomal triglyceride transfer protein (MTP, gene *Mttp*) is critical for the transport of lipids via apolipoprotein B (apoB)-containing lipoprotein assembly and secretion.^1^ This protein, which is abundant in the endoplasmic reticulum (ER) in hepatocytes and enterocytes, is believed to bind and transfer several lipids to apoB and to aid in lipoprotein assembly and secretion.^2^^,^^3^^,^^4^ Beyond hepatocytes and enterocytes, MTP is found in other cells that do not produce lipoproteins. For example, MTP supports the maturation of CD1 proteins in NKT cells^5^ and regulates adipose triglyceride lipase activity in adipocytes.^6^ Shoulders et al. reported the expression of *Mttp* mRNA in the testis in 1993^7^; however, MTP’s role in the testis has not yet been elucidated. Therefore, the aim of this study was to uncover the role of MTP in steroidogenesis in Leydig cells.

The process of steroidogenesis begins with the storage of neutral lipids, including cholesteryl esters and triglycerides, within cytosolic lipid droplets that serve as an intracellular reservoir of steroidogenic substrates. Upon luteinizing hormone (LH) stimulation of Leydig cells, cholesterol is mobilized through coordinated lipolysis and intracellular trafficking to the mitochondria. In the inner mitochondrial membrane (IMM), cholesterol is converted to pregnenolone by cytochrome P450 family 11 subfamily A member 1 (P450scc, gene *Cyp11a1*), representing the first and rate-limiting step in steroid hormone biosynthesis. Pregnenolone is subsequently transferred to the ER, where sequential enzymatic reactions mediated by 3β-hydroxysteroid dehydrogenase (3β-Hsd1) and cytochrome P450 enzymes produce testosterone.^8^

Steroidogenesis in Leydig cells is regulated by both acute and chronic responses. The acute response includes the receptor-mediated activation of a phosphorylation cascade facilitating cholesterol transport to the outer mitochondrial membrane (OMM) for subsequent hormone synthesis.^9^ In ovarian and adrenal cells, during the acute response, cholesterol is mobilized from lipid droplets to mitochondria for steroidogenesis. Subsequently, these cholesterol reserves are replenished with plasma cholesterol through lipoprotein uptake. In MA-10 and primary Leydig cells (PLCs), in addition to lipid droplets, cholesterol for acute steroidogenesis is also mobilized from the plasma membrane. De novo-synthesized cholesterol is used for subsequent steroid production.^10^^,^^11^^,^^12^ During chronic response, increased transcription of steroidogenic genes leads to overall enhancement of steroid synthetic capacity.^12^ Because Leydig cells rely largely on intracellular lipid pools and *de novo* cholesterol synthesis rather than plasma lipoprotein uptake, tight regulation of lipid storage, synthesis, mobilization, and trafficking is essential to sustain steroidogenic flux. Thus, maintenance of lipid homeostasis directly determines cholesterol availability for steroidogenesis and is a critical determinant of Leydig cell endocrine function.

Diminished serum testosterone or hypogonadism affects 2–13% of middle-aged to older men, whereas this rate is higher in men ≥45 years of age (38.7%) and individuals with diabetes or obesity (∼50%).^13^^,^^14^^,^^15^ Male hypogonadism is associated with several metabolic and physiological disorders, including diminished bone density, muscle mass, obesity, cardiovascular disease, and sexual functions.^16^ Administration of high testosterone concentrations eventually suppresses LH-mediated steroidogenesis and is not an effective treatment modality.^17^^,^^18^ Furthermore, clinical studies have suggested that high testosterone might potentially be linked to cardiovascular disease and cancer.^19^^,^^20^^,^^21^^,^^22^ Therefore, attempts are underway to modulate endogenous testosterone synthesis to avoid exogenous supplementation with large doses. Chung et al. have reported increased testosterone production in aging populations after enhancing translocator protein (TSPO) activity in the OMM.^23^ Uncovering additional mechanisms that regulate endogenous testosterone production might aid in hypogonadism treatment by modulating endogenous regulatory mechanisms.

Given the central importance of lipid homeostasis in Leydig cell steroidogenesis, proteins that regulate intracellular lipid handling and cholesterol biosynthesis are likely to play pivotal roles in controlling testosterone production. Sterol regulatory element-binding protein 2 (SREBP2) is a master transcriptional regulator of cholesterol biosynthesis and intracellular sterol balance. However, the role of SREBP2 and upstream modulators of SREBP2 activity in Leydig cells remains poorly defined.

In this study, we tested the hypothesis that MTP modulates Leydig cell steroidogenic capacity by regulating lipid homeostasis. Using complementary genetic, pharmacological, transcriptomic, and epigenomic approaches, we demonstrate that MTP is expressed in Leydig cells and that its deficiency enhances steroidogenesis by increasing SREBP2 expression, activity, and chromatin accessibility at key cholesterol and steroidogenic gene loci. These findings uncover a previously unrecognized role for MTP as a negative regulator of endogenous steroid hormone production through control of intracellular cholesterol metabolism and transcriptional networks in Leydig cells.

## Results

### MTP is present in Leydig cells of the testis

**Figure 1:**
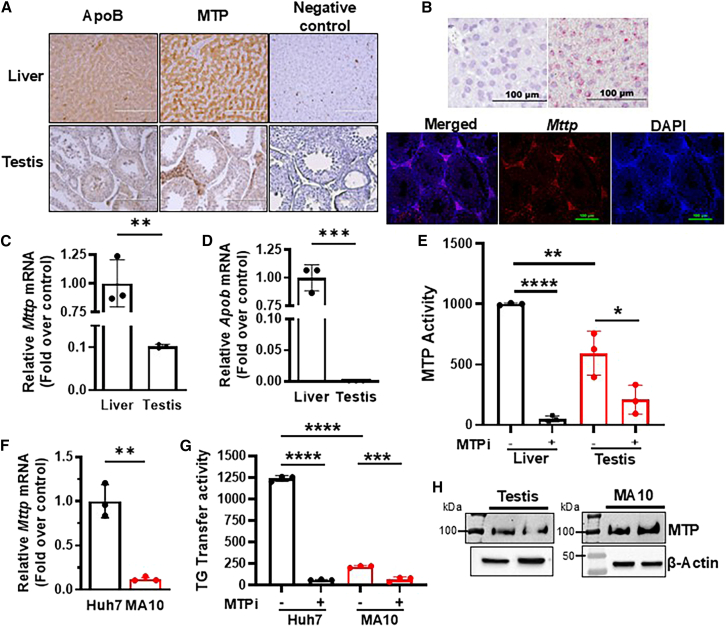
MTP is expressed in Leydig cells of the testis (A) Normal human liver (top) and testis (bottom) slides were used for immunohistochemistry. Primary antibody was omitted for the negative control. Scale bars, 200 μm. (B) *In situ* hybridization of *Mttp* mRNA in paraffinized sections (4 μm) of 3-month-old male mouse liver (upper) and testis (lower) with RNAScope. Liver sections were counterstained with hematoxylin for bright-field imaging with a negative control probe (left) and *Mttp* mRNA probe (right). Testis sections (bottom) were stained with MTP-specific probes, counterstained with DAPI, and merged (bottom). Fluorescence was imaged at 488 nm excitation and 561 nm emission wavelengths. (C and D) *Mttp* and *Apob* mRNA levels were measured in the testis of chow-fed 3-month-old male mice. Levels in the liver were normalized to 1. Mean ± SD (*n* = 3); ∗∗*p* < 0.01 and ∗∗∗*p* < 0.001, unpaired *t* test. Representative of three independent experiments. (E) Liver and testis lysates were assayed for the triglyceride transfer activity of MTP in the presence and absence of 0.5 μM lomitapide (MTP inhibitor, MTPi). Mean ± SD (*n* = 3); ∗, ∗∗, and ∗∗∗∗ refer to *p* < 0.05, 0.01, and 0.0001, respectively, one-way ANOVA. Representative of three independent experiments. (F) *Mttp* mRNA levels were measured in Huh7 and MA-10 cells after 48 h of culture. They were normalized to 18S levels and are represented as fold with respect to Huh7 cells. Mean ± SD (*n* = 3); ∗∗*p* < 0.01, unpaired *t* test. (G) Human hepatoma Huh7 (black) and mouse Leydig MA-10 (red) cells were treated or not with 0.5 μM lomitapide (MTPi) for 16 h. Cells were collected to measure triglyceride transfer activity. Mean ± SD (*n* = 3); ∗∗∗*p* < 0.001 and ∗∗∗∗*p* < 0.0001, one-way ANOVA followed by multiple comparisons. (H) Testis and MA-10 lysates (500 μg protein) were used to immunoprecipitate MTP using mouse anti-MTP and separated on gels. MTP was visualized after immunoblotting with anti-MTP and then with Veriblot secondary antibodies. Crude homogenates (15 μg) were used to detect β-actin in blots.

After *Mttp* mRNA was observed in human and mouse testis,^7^ global RNA and single-cell RNA sequencing data demonstrated the presence of *Mttp* mRNA in various cells of the testis.^24^^,^^25^^,^^26^ In addition, proteomics of Leydig cells has indicated that MTP protein is present and remains unchanged under forskolin-stimulated versus non-stimulated conditions.^27^ To determine whether the testis expresses MTP and apoB proteins, we performed immunohistochemistry in human testis sections. We verified the expression of apoB and MTP in human liver sections as positive controls. Antibodies to both apoB and MTP showed diffuse staining in the liver, and some areas showed punctate labeling consistent with the known expression of apoB and MTP in hepatocytes (Figure 1A, top). In contrast, the interstitial or Leydig cells of the human testis showed intense MTP staining, whereas apoB was scarcely detectable (Figure 1A, bottom). These studies indicated that human Leydig cells express MTP.

To uncover the role of MTP in Leydig cells, we extended studies to wildtype (WT) mice. First, *in situ* mRNA hybridization revealed *Mttp* mRNA in liver sections as punctate red labeling (Figure 1B, top right). In the mouse testis, *Mttp* mRNA was detected primarily in Leydig cells (Figure 1B, bottom middle). Second, we compared *Mttp* and *ApoB* mRNA levels in the liver and testis in mice. The *Mttp* mRNA levels were ∼10-fold lower (Figure 1C) and the *ApoB* mRNA levels were ∼10^5^-fold lower (Figure 1D) in the testis than in the liver. MTP’s triglyceride transfer activity was also significantly lower in the testis than in the liver (Figure 1E). In both tissues, MTP activity was significantly reduced by lomitapide, a specific MTP inhibitor (MTPi).^28^ These studies indicated the presence of *Mttp* mRNA and protein activity in the mouse testis.

Because immunohistochemistry in the human testis and *in situ* hybridization in the mouse testis showed intense staining in Leydig cells, we obtained the immortalized mouse Leydig cell line MA-10^29^ and compared the expression of *Mttp* in these cells versus human hepatoma Huh7 cells. The *Mttp* mRNA levels in MA-10 cells were ∼10% those in Huh7 cells (Figure 1F). In agreement with the mRNA levels, the triglyceride transfer activity was significantly lower in MA-10 than in Huh7 cell lysates and further reduced after MTPi treatment in both cell lines (Figure 1G). Next, we detected MTP protein in mouse testis and MA-10 cells after immunoprecipitation (Figure 1H). Therefore, *Mttp* mRNA, protein, and activity were observed in MA-10 cells.

### MTP deficiency increases steroid production in Leydig cells

**Figure 2:**
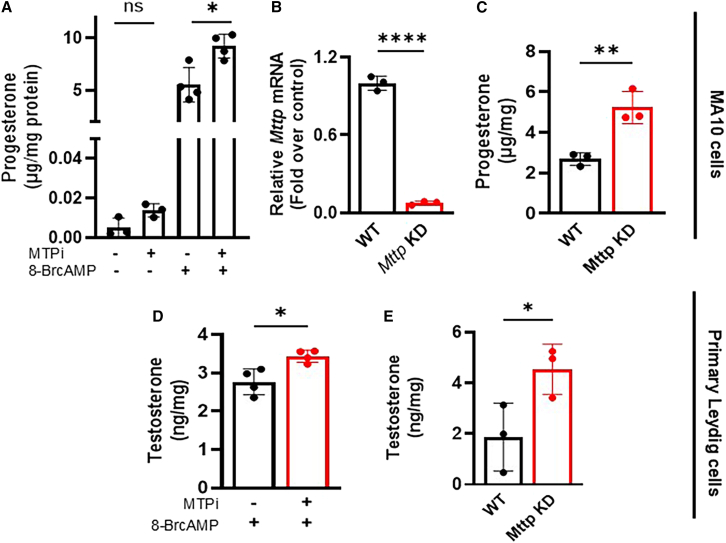
Effect of MTP inhibition on steroid production in MA-10 and primary Leydig cells (A) MA-10 cells were cultured for 16 h in the presence or absence of 1 μM MTPi, washed, and treated or not with 0.5 mM 8-BrcAMP for 3 h in serum-free F12 medium with or without MTPi. Progesterone was measured using a kit. Mean ± SD (*n* = 3); representative of three independent experiments; ns, not significant, ∗*p* < 0.05, one-way ANOVA followed by multiple comparisons. (B and C) MA-10 cells were transfected with sgRNA and Cas9 via electroporation. After 3 days, cells were collected to measure *Mttp* mRNA levels (B). Another set of cells was used to measure secreted progesterone and cellular protein levels after stimulation with 8-BrcAMP for 3 h (C). Mean ± SD (*n* = 3); representative of three independent experiments. ∗∗*p* < 0.01 and ∗∗∗∗*p* < 0.0001, unpaired *t* test. (D) Primary Leydig cells were isolated from 4-month-old mice and cultured for 72 h. Cells were further treated with 1 μM of lomitapide for 16 h in serum-containing F12 medium and 3 additional hours in serum-free medium in the presence of 8-BrcAMP. Testosterone was measured from the media and normalized with total protein. Mean ± SD (*n* = 3); ∗*p* < 0.05, unpaired *t* test. (E) Cultured primary Leydig cells were electroporated with *Mttp* sgRNA and Cas9 as described in 2B. After 48 h, cells were stimulated with 8-BrcAMP for 3 h, and the media were collected for testosterone measurement. Mean ± SD (*n* = 3); ∗*p* < 0.05, unpaired *t* test.

The major function of Leydig cells is sex hormone production. MA-10 cells synthesize progesterone instead of testosterone as the main steroid hormone.^16^ To examine the role of MTP in steroid production, MA-10 cells were treated or not with MTPi for 16 h, then treated with or without 8-BrcAMP for 3 h to stimulate progesterone synthesis. Under basal conditions, MA-10 cells secreted very low levels of progesterone (Figure 2A). The secretion of progesterone increased by ∼500-fold in control cells after 8-BrcAMP treatment (Figure 2A), consistent with findings from other studies.^30^^,^^31^ Unexpectedly, MTPi further enhanced progesterone secretion by ∼1.5- to 2-fold in stimulated cells (Figure 2A). Furthermore, transient knockdown of *Mttp* mRNA by >90% with sgRNA-Cas9 (Figure 2B) significantly enhanced progesterone production by ∼2-fold (Figure 2C). These studies suggested that MTP deficiency significantly augments progesterone production.

MA-10 cells are a well-studied model for Leydig cell steroidogenesis; however, they are far from an accurate model of Leydig cell physiology. Therefore, we extended these studies to mouse PLCs. First, we studied the purity of cell preparation. Based on qPCR, PLC were 10-fold-enriched in *Cyp11a1* mRNA, a Leydig cell marker, compared to the testis (Figure S1A) and were significantly deficient in *Rhox5* (Sertoli cell marker) and *Dbil5* (germ cell marker) expression (Figure S1A). Second, a significant number of cells were 3β-Hsd1^+^ (Figure S1B). Third, these cells secreted more testosterone in response to 8-BrcAMP (Figure S1C). These studies indicated a successful culture of PLCs. Next, we studied the effect of MTP inhibition and knockdown on testosterone secretion in these cells (Figures 2D and 2E). Both MTP inhibition and sgRNA-mediated knockdown by ∼73% increased testosterone secretion compared to control cells.

**Figure 3:**
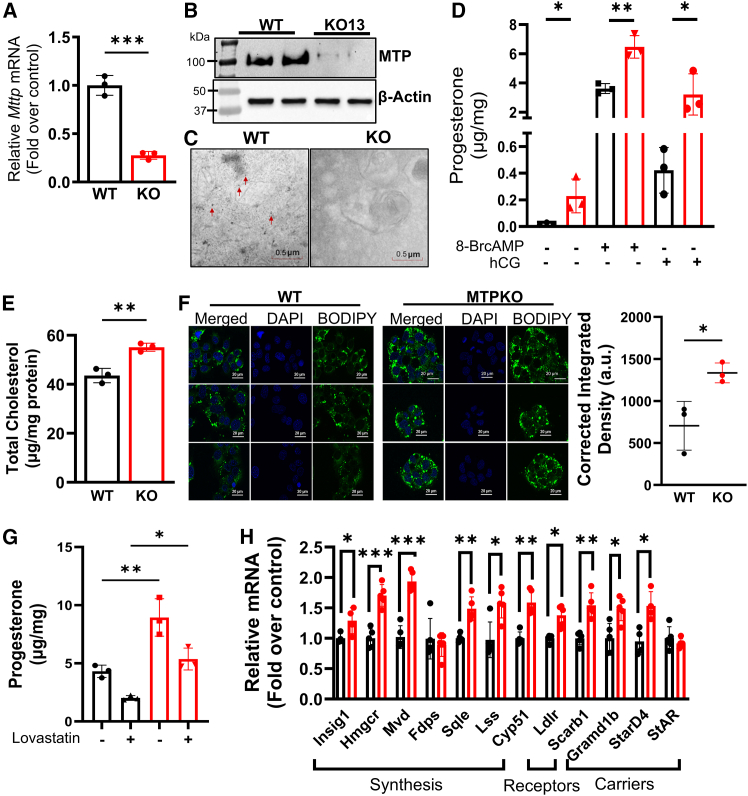
Increased cholesterol accumulation and steroid secretion in MTP KO cells (A) Cas9 and specific sgRNA were electroporated to establish MTP-deficient MA-10 clones. mRNA levels were measured in control and MTP-deficient cells. Mean ± SD (*n* = 3); ∗∗∗*p* < 0.001, Student’s *t* test. (B) MTP protein expression was detected by immunoprecipitation followed by immunoblotting with anti-MTP in lysates from WT MA-10 cells but not in the MTP KO cell clone. Representative of three independent experiments. (C) Gold immunoelectron microscopy of WT and KO cells with anti-MTP primary antibody followed by gold-labeled secondary antibody. Images were captured at 30,000× magnification. Black dots representing MTP expression indicated by red arrows are visible in WT cells (left) but not in KO cells (right). (D) Medium from 48 h cultures of WT and KO cells was used to measure amounts of progesterone secreted after the stimulation of the cells with 0.5 mM 8-BrcAMP or 20 ng/mL hCG for 3 h. Representative of three independent experiments. Mean ± SD (*n* = 3); ns, not significant; ∗*p* < 0.05 and ∗∗*p* < 0.01, unpaired *t* test. (E) Total lipids were extracted from cells with isopropanol, and total cholesterol levels were measured. Representative of three independent experiments. Mean ± SD (*n* = 3); ∗∗*p* < 0.01, unpaired *t* test. (F) WT and KO cells were cultured in 8-chambered slides for 48 h and stained for lipid droplets with BODIPY480/508 cholesterol and imaged at 488 nm and 561 nm excitation and emission filters, respectively, at 40× magnification (left). Fluorescence was quantified and plotted (right). Mean ± SD (*n* = 3); ∗*p* < 0.05, unpaired *t* test. (G) WT and KO cells were treated with or without 0.5 μM lovastatin for 16 h and subsequently treated with 8-BrcAMP for 3 h. Progesterone was measured in the medium. Mean ± SD (*n* = 3); ∗*p* < 0.05 and ∗∗*p* < 0.01, one-way ANOVA with multiple comparisons. (H) mRNA levels of cholesterol metabolism genes. Mean ± SD (*n* = 5); ∗*p* < 0.05, ∗∗*p* < 0.01, ∗∗∗*p* < 0.001, and ∗∗∗∗*p* < 0.0001, unpaired *t*-tests between WT (black) and KO (red) cells.

To investigate further the role of MTP deficiency in steroidogenesis, we generated an MTP KO MA-10 cell line (KO13) with CRISPR/Cas9 that had significantly lower levels of *Mttp* mRNA than control cells, as determined by qPCR (Figure 3A). In this clone, no appreciable amounts of MTP protein were detected after immunoprecipitation and immunoblotting (Figure 3B). Furthermore, by gold immunolabeling, we detected MTP in the ER of the WT MA-10 cells but not the KO cells (Figure 3C). These studies validated the establishment of MTP-deficient MA-10 cells. We next characterized cell growth and ER stress in this clone. KO and WT cells showed similar growth (Figure S2A). Furthermore, the selected ER stress markers CHOP and ATF6 were similar in KO and control cells (Figure S2B). These studies indicated that chronic MTP deficiency had little effect on cell growth and ER stress under these experimental conditions.

Next, we studied progesterone secretion in this clone under basal conditions and after stimulation. Steroid production increased by 4500% and 500% after stimulation with 8-BrcAMP and hCG, respectively, in WT cells (Figure 3D), in agreement with findings from previous studies.^9^^,^^32^ MA-10 cells exhibit lower responsiveness to hCG than the cell-permeable cAMP analog, because of lower LH/choriogonadotropin receptor (Lhcgr) activity.^33^^,^^34^ The amount of progesterone secreted by KO13 clone was 80% and 664% greater than that observed in WT cells after treatments with hCG and 8-BrcAMP, respectively (Figure 3D). Therefore, MTP inhibition, transient knockdown, and gene KO increased progesterone production in MA-10 cells.

### MTP ablation increases the expression of genes involved in cholesterol synthesis, lipoprotein receptors, and intracellular cholesterol trafficking to the mitochondria

Cholesterol is the substrate for steroidogenesis. Therefore, we hypothesized that the highly steroidogenic MTP-deficient cells would have greater cholesterol levels than normal Leydig cells. Indeed, the MTP KO cells had significantly higher total cholesterol levels than the WT cells (Figure 3E). The increased cholesterol was further evident when the lipid droplets were stained with BODIPY-Cholesterol (Figure 3F). However, the total and free cholesterol levels in the ER did not differ between WT and KO cells (Figure S2C). We then focused on mechanisms underlying the elevated cholesterol levels in MTP-deficient cells. Steroidogenic cells obtain cholesterol via two pathways: *de novo* synthesis and uptake of lipoproteins.^8^ To determine whether cholesterol biosynthesis might be required for enhanced progesterone secretion by MTP-deficient cells, we treated WT and KO13 cells with lovastatin, a competitive inhibitor of HMG-CoA reductase that reduces cholesterol biosynthesis.^35^ Lovastatin significantly inhibited progesterone secretion in both WT and KO cells, thus indicating that cholesterol biosynthesis contributes to enhanced progesterone production (Figure 3G). We also observed significantly greater mRNA levels of genes involved in cholesterol biosynthesis, including Insulin-induced gene 1 *(Insig1),* hydroxy-3-methylglutaryl-coenzyme A reductase (*Hmgcr),* mevalonate diphosphate decarboxylase *(Mvd),* squalene epoxidase *(Sqle),* lanosterol synthase *(Lss)*, and cytochrome P450 14α-sterol demethylase (*Cyp51*), in MTP KO cells treated with 8-BrcAMP than in WT cells (Figure 3H). Moreover, MTP KO cells had higher mRNA levels of LDL receptor (*Ldlr*) and Scavenger receptor class B type 1 (SRB1, HDL receptor, gene *Scarb1*) than the control cells (Figure 3H). Thus, MTP-deficient cells showed increased expression of genes in cholesterol biosynthesis and lipoprotein uptake.

A critical step in steroidogenesis is the cleavage of the aliphatic chain of cholesterol in the IMM^16^ after cholesterol is transported from different sub-cellular organelles to mitochondria by specialized carrier proteins. StarD4 and StarD1 (also known as StAR) transport cholesterol for steroidogenesis.^36^^,^^37^ STARD4 functions as a general non-vesicular cholesterol transporter and transports cholesterol from different organelles to mitochondria, whereas STARD1, commonly known as StAR, acts at the OMM to promote cholesterol transfer from the OMM to IMM. Although StAR mRNA levels did not differ, *Stard4* mRNA levels were significantly higher in KO than in control cells (Figure 3H). Recently, Aster B protein (gene, *Gramd1b*)^38^ in mitochondria-ER contact sites has been shown to mediate cholesterol transport from the ER to the mitochondria during steroidogenesis.^39^ *Gramd1b* mRNA levels were significantly higher in MTP KO than WT cells after stimulation (Figure 3H). Therefore, MTP deficiency increases the expression of proteins in intra-organelle cholesterol transport. In summary, these studies indicated that MTP deficiency augments the expression of genes in lipoprotein uptake, cholesterol biosynthesis, and intracellular transport to mitochondria.

### *Mttp* gene ablation increases steroidogenesis in MA-10 cells

**Figure 4:**
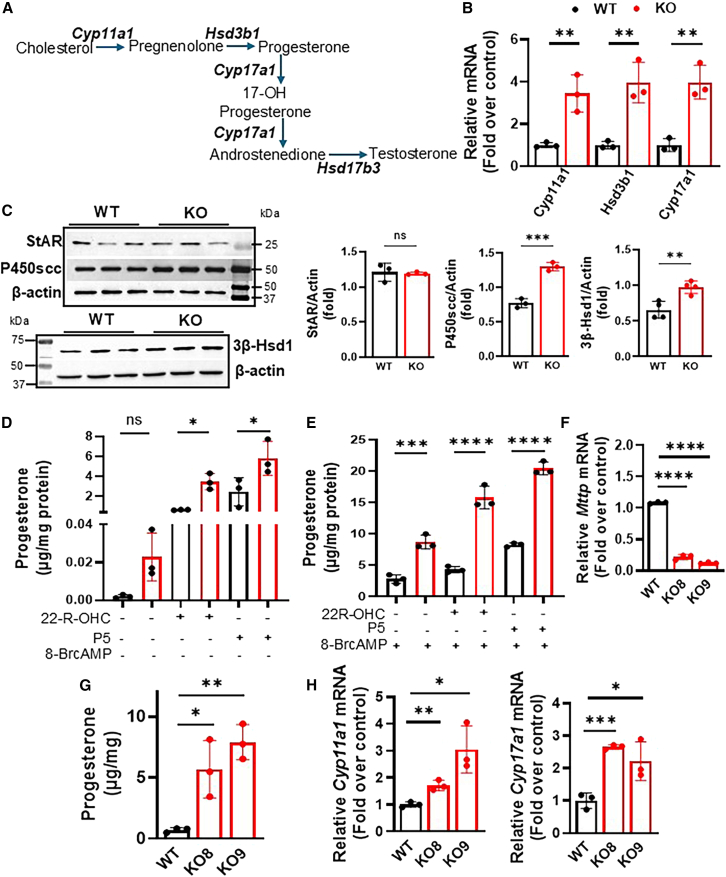
*Mttp* gene ablation upregulates the steroidogenic pathway (A) Schematic diagram of the testosterone synthesis pathway in mouse Leydig cells. Cholesterol is converted to pregnenolone by the P450scc enzyme, which resides in the IMM. Pregnenolone diffuses out of the mitochondria and is oxidized in the smooth ER by 3β-Hsd1, thus producing progesterone. Cyp17 group of oxidative enzymes then produces a variety of steroids. (B) mRNA levels of different steroidogenic genes in WT and KO cells. Mean ± SD (*n* = 3); ∗∗*p* < 0.01, unpaired *t* test. (C) Immunoblots showing steroidogenic Star, P450scc, and 3β-Hsd1 protein levels in 8-BrcAMP-induced cells (left). Quantification of the blots (right). Representative of three independent experiments. Mean ± SD (*n* = 3); ns, not significant, ∗∗*p* < 0.01 and ∗∗∗*p* < 0.001, unpaired *t* test. (D and E) Progesterone levels in the medium of WT (black) and KO (red) were treated with different substrates for 3 h. Additionally, they were treated (D) or not (E) with 0.5 mM 8-BrcAMP for 3 h. Representative of three independent experiments. Mean ± SD (*n* = 3); ∗*p* < 0.05, ∗∗∗*p* < 0.001, and ∗∗∗∗*p* < 0.0001, one-way ANOVA followed by multiple comparisons. (F) *Mttp* mRNA levels in two additional MTP KO clones. Mean ± SD (*n* = 3); ∗∗∗∗*p* < 0.0001, one-way ANOVA followed by multiple comparisons. (G) Progesterone secretion in MTP-deficient clones cultured for 48 h and treated with 0.5 mM 8-BrcAMP for 3 h. Representative of three independent experiments. Mean ± SD (*n* = 3); ∗*p* < 0.05 and ∗∗*p* < 0.01, one-way ANOVA followed by multiple comparisons. (H) mRNA levels of steroidogenic genes in the MTP KO clones after 8-BrcAMP stimulation. Representative of three independent experiments. Mean ± SD (*n* = 3); ∗, ∗∗, and ∗∗∗ refer to *p* < 0.05, 0.01, and 0.001, respectively, multiple t-tests.

Next, we examined the progesterone synthesis pathway in MTP-deficient cells. In the IMM, cytochrome P450 side-chain cleavage enzyme (P450scc, gene *Cyp11a1*) catalyzes the conversion of cholesterol to pregnenolone. Subsequently, the 3β-hydroxysteroid dehydrogenase/isomerase type 1 (3β-Hsd1, gene *Hsd3b1*) enzyme converts pregnenolone to progesterone in smooth ER (Figure 4A). Pregnenolone can be converted to other androgens, such as testosterone, by cytochrome P450 17A1 (Cyp17a1) and Hsd17b3 in the ER. Because Cyp17a1 protein is non-functional in MA-10 cells, progesterone is secreted instead of testosterone as the main steroid hormone.^16^ In KO cells, we observed greater mRNA levels of *Cyp11a1*, *Hsd3b1*, and *Cyp17a1* in KO cells compared to WT cells (Figure 4B), and we additionally detected significantly higher protein levels of P450scc and 3β-Hsd1 by immunoblotting (Figure 4C). In agreement with the absence of changes in mRNA levels (Figure 4B), the protein levels of StAR did not differ in KO and WT cells (Figure 4C). This was further validated under both stimulated and unstimulated conditions. *Cyp11a1* mRNA and protein levels increased in the KO cells under both conditions (Figure S2E). StAR levels were undetectable in unstimulated cells and increased to similar levels in both WT and KO cells (Figure S2E). These studies demonstrated that MTP deficiency increases mRNA levels of key enzymes in steroidogenesis in a StAR-independent manner.

Additionally, we examined changes in the activities of P450scc and 3β-Hsd1 enzymes. To measure the activities of these enzymes *in vivo,* their water-soluble substrates, 22-R-hydroxycholesterol and pregnenolone, respectively, were provided. Because these substrates are readily taken up by cells, the amounts of progesterone produced correlate with enzyme activity.^40^ Progesterone secretion significantly increased in both WT and KO cells after supplementation with both these substrates under basal (Figure 4D) and stimulated (Figure 4E) conditions, thus indicating higher activities of P450scc and 3β-Hsd1 in KO cells. Therefore, MTP deficiency increases the expression and activity of enzymes in the steroidogenic pathway.

We considered that the KO13 clone might uniquely augment progesterone production. To test this possibility, we established two more MTP-deficient clones, KO8 and KO9 (Figure 4F). Both clones secreted significantly higher amounts of progesterone than WT cells under stimulated conditions (Figure 4G). Moreover, these cells showed higher mRNA levels of *Cyp11a1* and *Cyp17a1* than observed in WT cells (Figure 4H). These studies indicated that MTP deficiency enhances progesterone secretion in multiple clones.

### Upregulation of the steroidogenic pathway in MTP KO cells

**Figure 5:**
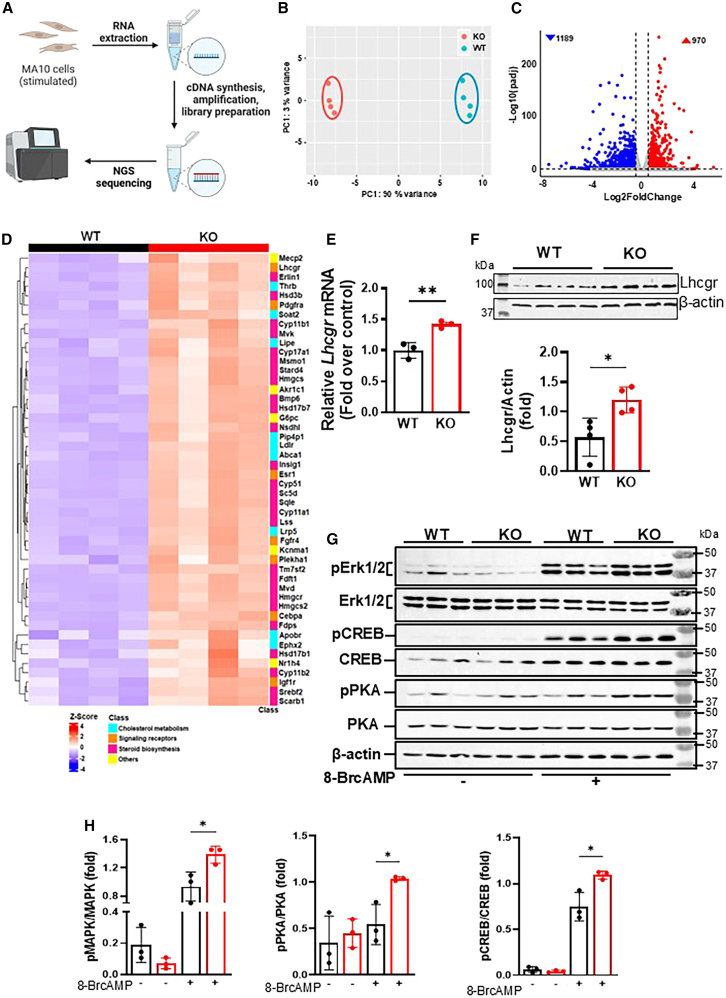
MTP deficiency enhances steroidogenic gene expression and increases Lhcgr signaling in MA-10 cells (A) Schematic diagram depicts the RNA-seq workflow. Briefly, 48 h old cultures of MA-10 cells were stimulated with 8-BrcAMP, and RNA was isolated. RNA purity and integrity were assessed. mRNAs were isolated with polyU columns and used for cDNA synthesis and library preparations for next-generation sequencing. (B) Principal component analysis of the RNA-seq data shows well-separated clusters of mRNA levels in WT and KO cells. (C) Volcano plot shows differentially regulated genes in transcriptomic analysis of WT and KO cells. (D) Heatmap showing upregulated genes in steroid metabolism in the MTP KO cells. (E and F) Upregulation of *Lhcgr* mRNA (E) and protein (F) levels [immunoblots (top) and quantifications (bottom)] in KO cells. Mean ± SD (*n* = 3); ∗*p* < 0.05 and ∗∗*p* < 0.01, unpaired *t* test. (G and H) Immunoblots show the phosphorylation of PKA, MAPK, and CREB in WT and KO cells treated or not with 8-BrcAMP for 20 min (G) and their quantification (H), presented as means ± SD (*n* = 3); ∗*p* < 0.05, unpaired *t* test. Representative of three independent experiments.

To gain a comprehensive understanding of gene expression changes associated with steroidogenesis, we performed RNA-Seq in WT and KO cells (Figure 5A). Principal component analysis revealed two distinct clusters for the WT and KO groups with no overlap, thus indicating a significant difference between them (Figure 5B). A correlation heatmap displayed high consistency among the replicates within each group, which were clustered together (Figure S3A). According to differential gene expression analysis, among 2159 differentially expressed genes, 1189 were downregulated, and 970 were upregulated (Figure 5C). GO and KEGG pathway analyses revealed that the sterol metabolism and steroid biosynthesis pathways were highly enriched in KO cells (Figures S3B and S3C). However, it had no effect on the ER stress pathway (Figure S3D). These transcriptomic and bioinformatics analyses also indicated that MTP deficiency enhances cholesterol and steroid biogenesis pathways in MA-10 cells, thus confirming our earlier observations.

RNA-seq also confirmed the elevated expression of *Cyp11a1* and *Hsd3b1* in KO cells (Figure 5D), as previously observed (Figures 4B and 4C). Beyond these enzymes, we observed increases in *Lhcgr* mRNA (Figure 5D) and confirmed with qRT-PCR and immunoblotting (Figures 5E and 5F). After ligand binding, Lhcgr activates various kinases by phosphorylation and consequently enhances gene expression. Therefore, we asked whether these phosphorylation cascades might be enhanced in KO cells. Through western blotting, we observed an increase in the phosphorylation of Erk1/2, PKA, and CREB in KO cells stimulated with 8-BrcAMP (Figures 5G and 5H). These results demonstrated increased Lhcgr expression and signaling in MTP-deficient MA-10 cells.

The above studies indicated increased progesterone synthesis in MTP KO cells. Steroid biosynthesis requires substantial energy, which can be derived from enhanced fatty acid oxidation and glycolysis. Fatty acids are stored in lipid droplets, which are mobilized after the activation of hormone-sensitive lipase (HSL). MTP KO cells showed greater lipolysis than WT cells, and this activity was significantly inhibited by an HSL inhibitor (Figure S4A). These findings, therefore, indicated that the KO cells had elevated HSL activity. In the MA-10 cell line, glycolysis provides a major source of ATP for steroidogenesis).^41^ In addition, energy for steroid synthesis is derived from the oxidative phosphorylation of fatty acids.^42^^,^^43^^,^^44^^,^^45^ We observed significantly greater oxygen consumption rate (OCR) in KO cells than WT cells (Figure S4B). We also observed greater glycolysis rates, as measured by extracellular acidification rates in KO cells than WT cells (Figure S4C). However, we did not find significant differences in the mitochondrial complex proteins (Figure S4D), thus indicating enhanced mitochondrial activity with no change in mitochondrial mass in KO than WT cells. These studies demonstrated that KO cells have enhanced fatty acid oxidation and glycolytic rates. In brief, MTP deficiency appears to upregulate various physiological processes to support enhanced steroid biosynthesis, including cholesterol uptake, mobilization, and synthesis, as well as the upregulation of enzymes involved in steroid biosynthesis, fatty acid oxidation, and glycolysis.

### MTP regulates steroidogenesis by increasing the expression and activity of SREBP2

Steroidogenesis in gonads and adrenal glands is regulated predominantly by the Steroidogenic factor-1 (SF-1, gene *Nr5a1*) transcription factor.^46^^,^^47^^,^^48^ Our RNA-Seq data showed no change in mRNA levels of *Nr5a1*. Furthermore, qRT-PCR analysis also showed that *Nr5a1* mRNA levels did not differ between MTP KO and WT cells (Figure S4E). Additionally, the total and phosphorylated SF-1 protein levels were similar in both cell types (Figure S4F). These studies indicated that the elevated steroidogenesis in MTP-deficient cells might not involve increased expression and phosphorylation of SF-1.

**Figure 6:**
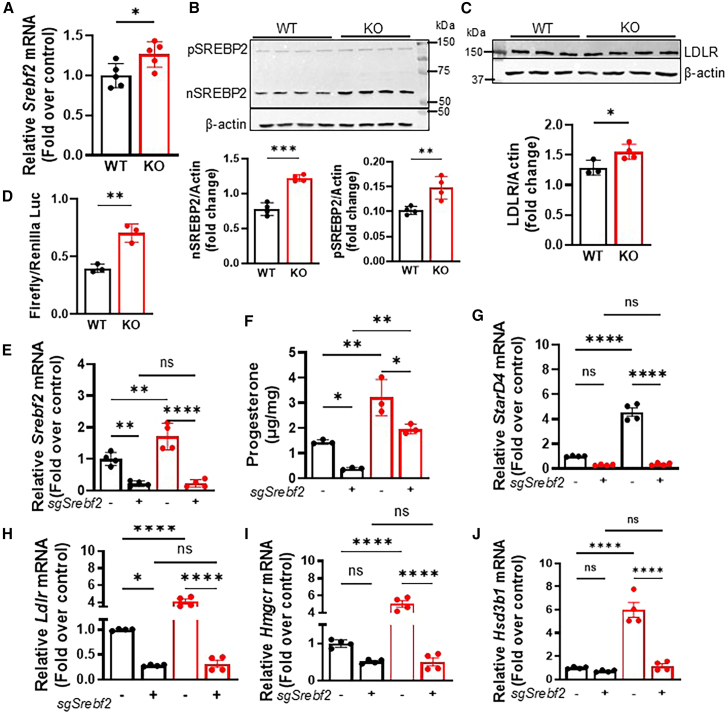
MTP ablation increases SREBP2 expression and activity (A) qPCR of *Srebf2* mRNA levels in WT and KO cells after 8-BrcAMP stimulation. Representative of three independent experiments. Mean ± SD (*n* = 5); ∗*p* < 0.05, unpaired *t* test. (B and C) Western blots of 8-BrcAMP stimulated WT and KO cell lysates probed with anti-SREBP2 (B) and anti-LDLR (C). The blots were re-probed with anti-β-actin for normalization. Quantification (middle and right) of the blots is presented normalized to β-actin, as mean ± SD (*n* = 4). ∗*p* < 0.05, ∗∗*p* < 0.01, and ∗∗∗*p* < 0.001, unpaired Student’s *t* test. pSREBP2, precursor form of SREBP2; nSREBP2, nuclear form of SREBP2. (D) Luciferase activity in WT and KO cells. Mean ± SD (*n* = 3). ∗∗*p* < 0.01, unpaired Student’s *t* test. (E) *Srebf2* mRNA levels in WT (black) and KO (red) cells after electroporation with specific sgRNA-Cas9. Mean ± SD (*n* = 4). ∗∗*p* < 0.01 and ∗∗∗∗*p* < 0.0001, one-way ANOVA followed by multiple comparisons. (F) Progesterone levels were significantly reduced after Srebf2 knockdown in both WT and KO cells compared to their respective control cells not treated with sgRNA. Mean ± SD (*n* = 3). ∗*p* < 0.05 and ∗∗*p* < 0.01, one-way ANOVA followed by multiple comparisons. (G–I) mRNA levels of the SREBP2 target genes *Stard4*, *LdIr* and *Hmgcr*, measured by qPCR. Mean ± SD, *n* = 4. ∗*p* < 0.05 and ∗∗∗∗*p* < 0.0001, one-way ANOVA followed by multiple comparisons. (J) mRNA level of the steroidogenic gene *Hsd3b1*, measured by qPCR. Representative of 3 independent experiments. Mean ± SD (*n* = 4). ∗∗∗∗*p* < 0.0001, one-way ANOVA followed by multiple comparisons.

We next examined SREBP2, a known regulator of cholesterol metabolism.^31^^,^^46^ We observed greater mRNA of *Srebf2* in RNA-Seq (Figure 5D), as confirmed by qRT-PCR and immunoblotting (Figures 6A, 6B, and S4G) in KO than WT cells. Furthermore, the mRNA and protein levels of *Ldlr*, a target of SREBP2, were higher in KO cells (Figures 3H and 6C). In addition, we measured SREBP2 activity with dual luciferase assays. SREBP2 activity was significantly higher in KO than in WT cells (Figure 6D). Because these studies indicated increased expression and activity of SREBP2 in MTP KO cells, we hypothesized that SREBP2 might play a role in the upregulation of steroidogenesis in MTP-deficient cells. To test this possibility, we knocked down *Srebf2* gene expression by ∼85–90% in both WT and MTP KO MA-10 cells using specific sgRNA and Cas9 (Figure 6E). *Srebf2* sgRNA significantly attenuated progesterone secretion in WT and KO cells (Figure 6F). Furthermore, the mRNA levels of several *Srebf2* target genes in cholesterol metabolism, *Stard4*, *Ldlr,* and *Hmgcr*, significantly decreased in WT and MTP-deficient cells after *Srebf2* knockdown (Figures 6G–6I). These studies indicated that *sgSrebf2* significantly decreased the expression of SREBP2 and its downstream target genes in cholesterol biosynthesis.

Next, we examined the expression of genes in the steroidogenesis pathway. Unexpectedly, the mRNA levels of the steroidogenic gene *Hsd3b1* were significantly diminished in the *Srebf2* KO cells (Figure 6J), thereby indicating that SREBP2 also reduces the expression of genes in steroidogenesis. SREBP2 might, therefore, be an important regulator of steroidogenesis in MA-10 cells. We then asked whether MTP regulates progesterone secretion mainly by regulating SREBP2. To address this, we first reduced *Srebf2* expression using specific sgRNA and then treated cells with MTPi (Figure S4H). MTPi increased progesterone secretion in control and sgRNA-treated cells (Figure S3H), indicating that MTP may also regulate pathways independent of SREBP2 to regulate progesterone secretion.

### Changes in chromatin accessibility of various TFs in MTP-deficient MA-10 cells

**Figure 7:**
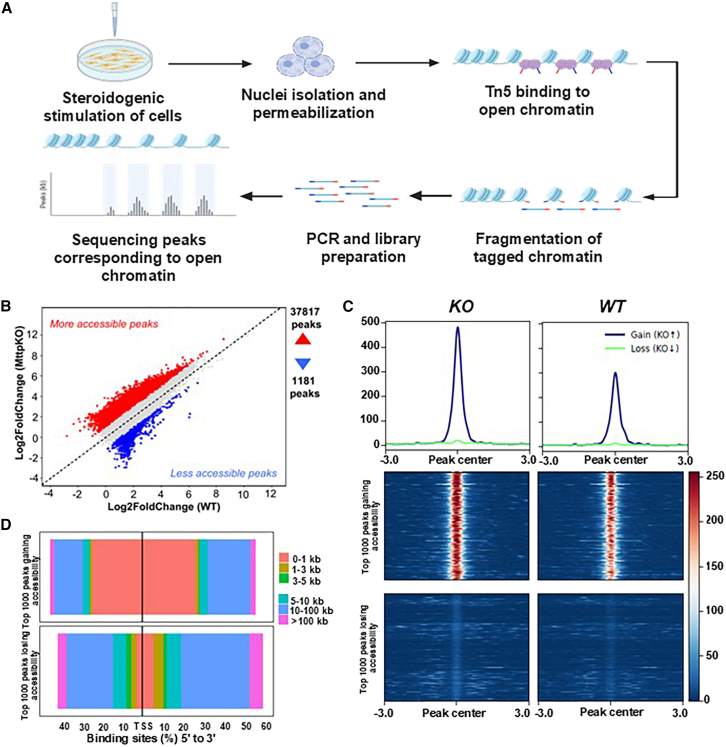
Global chromatin accessibility changes due to MTP deficiency in MA-10 cells (A) Schematic overview of the bulk ATAC-seq workflow. Cells were stimulated, nuclei were isolated and permeabilized, Tn5 transposase was used to insert sequencing adapters into accessible chromatin, and library amplification, sequencing, and identification of open chromatin regions were performed. (B) Scatterplot of changes in differential chromatin accessibility in KO cells versus WT MA-10 cells. Each dot represents a genomic peak; red denotes significantly more accessible peaks in KO cells, whereas blue indicates less accessible peaks relative to WT. Numbers indicate total differential peaks in each direction. (C) Aggregate accessibility profiles (top) and heatmaps (bottom) for the top 1,000 peaks with greater accessibility (gain, KO↑; blue) and the top 1,000 peaks with less accessibility (loss, KO↓; green) in KO than WT cells. Peaks with greater accessibility exhibited a strong and sharp enrichment at the peak center in KO relative to WT, whereas peaks classified as having less accessibility showed minimal or no discernible differences between conditions. Signal intensities are normalized read counts ±3 kb around the peak center. (D) Genomic distribution of differential peaks relative to TSS. Bar plots indicate the percentage of gained (top) or lost (bottom) peaks distributed across genomic distances from the nearest TSS. Gain of accessibility was highest within ± 1 kb of the TSS.

To delineate further how MTP deficiency affects gene expression, we performed ATAC-Seq (Figure 7A). A total of 37,817 peaks showed gain of accessibility, whereas 1,187 genes showed loss of accessibility in MTP KO cells versus WT cells (Figure 7B). To evaluate chromatin accessibility, we created aggregate profiles and heatmaps based on consensus ATAC-seq peaks (±3 kb) (Figure S5A). Notably, MTP KO cells showed stronger accessibility signals than WT cells, as evidenced by their greater enrichment at the peak summits and broader signal distribution over flanking regions. These findings suggested greater chromatin accessibility in MTP KO cells than WT cells, and implied that MTP deletion causes broad modification of the chromatin accessibility landscape.

We analyzed the differential accessibility of the top 1000 peaks with the greatest gains or losses in MTP KO versus WT cells. Aggregate profiles and heatmaps revealed significant signal enrichment at the centers of the acquired peaks in KO cells, thus indicating markedly enhanced accessibility, but the top 1000 lost peaks showed little difference with respect to those in WT cells (Figure 7C). Genomic distribution analysis demonstrated that most acquired peaks (70.5%) were in promoter regions (Figure S5B). In contrast, the peaks with the greatest loss of accessibility were in distal intergenic regions (34.6%). Therefore, MTP loss largely induced chromatin opening at promoter regions, and loss of chromatin accessibility tended to occur at distal intergenic elements. Furthermore, we investigated the distance between differential peaks and transcription start sites (TSSs). Peaks showing gain of accessibility in KO cells were enriched near the TSS (0–1 kb and 1–3 kb), thus indicating promoter-centric augmentation (Figure 7D). In contrast, peaks with loss of accessibility were more widely distributed among distal elements, approximately 10–100 kb and >100 kb from the TSS. These findings suggested that MTP deletion caused broad chromatin opening at gene promoter regions, whereas decreased accessibility was limited primarily to distal intergenic areas.

**Figure 8:**
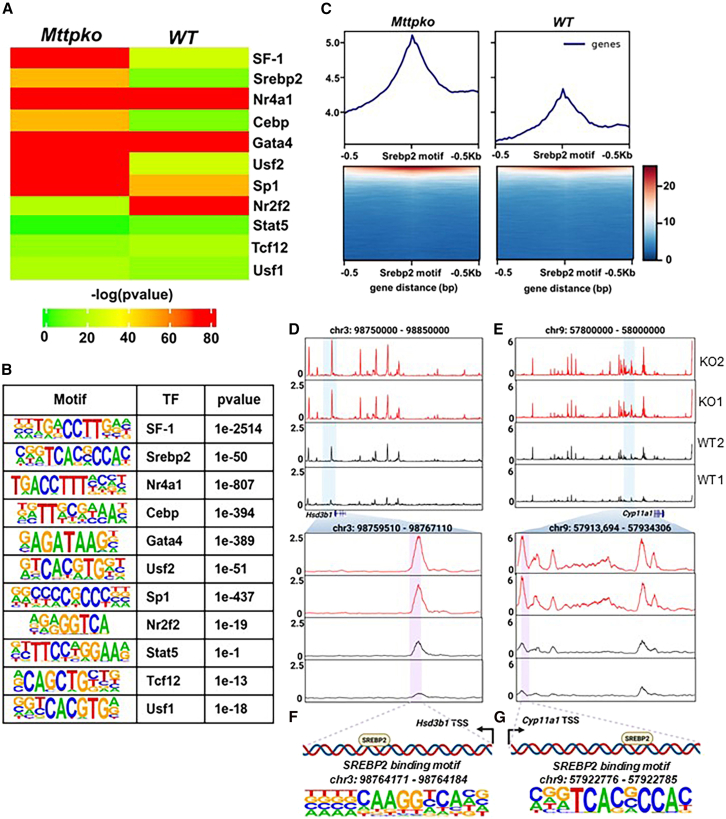
Enrichment in different TF binding motifs in MTP-deficient cells (A) Heatmap of transcription factor motif enrichment across KO and WT replicates. The most significantly enriched motifs are displayed, with the color scale representing –log_10_ (*p*-value). Notable enriched motifs in the KO cells. (B) Representative enrichment in DNA motifs within differential peaks of MTP KO cells. Binding motifs are annotated with their corresponding TF and enrichment *p*-value, highlighting enrichment in KO cells. (C) Aggregate accessibility profiles (top) and heatmaps (bottom) of ATAC-seq signals centered on consensus SREBP2 motifs (±0.5 kb) in KO and WT cells, showing greater accessibility around both motifs in KO than WT cells. (D–G) Genome-wide accessibility landscapes at specific gene loci. (D, E) Normalized ATAC-seq tracks across broad regions of chromosome 3 (*Hsd3b1* locus) and chromosome 9 (*Cyp11a1* locus), respectively, revealing overall chromatin accessibility differences between duplicate KO and WT measurements. Shaded blue areas indicate the gene loci containing the SREBP2 binding motif and are enlarged as pink shaded peaks to show the SREBP2 binding motif (G, H).

Furthermore, we searched across all differential peaks for specific enrichments in binding motifs for TFs known to regulate steroidogenesis in Leydig cells. We found enrichment in SREBP2, SF-1, CEBP, USF2, and SP1 binding motifs, and diminished enrichment in the NR2F2 binding motif in MTP KO cells (Figure 8A). The binding motifs of GATA4 and NR4A1 remained highly enriched in both the WT and KO cells (Figure 8A). Figure 8B lists the corresponding motifs and *p* values for various TFs. Subsequently, the accessibility profiles revealed significant enrichment in the accessibility of SREBP2 motifs in MTP KO cells versus WT cells (Figure 8C). These findings demonstrated that MTP deficiency increases the chromatin accessibility of important steroidogenic TFs to their binding motifs and might explain the activation of sterol metabolism and steroid biosynthesis regulatory networks.

To understand the regulation of enzymes in steroid and cholesterol biosynthesis, we examined the changes in chromatin accessibility around the TSSs in the promoters of *Hsd3b1, Cyp11a1* (Figures 8D–8G)*, Hmgcr*, and *Ldlr* (Figures S5C and S5D). Whereas chromatin accessible peaks in all these genes were found in WT cells, their greater accessibility was observed in KO cells (Figures 8D, 8E, S5C, and S5D). Analysis of peaks in the *Hsd3b1* and *Cyp11a1* genes revealed the presence of SREBP2 binding motifs in the enhanced peaks. In *Hsd3b1*, the SREBP2 binding motif (98,764,171–98,764,184 bp) was detected (Figure 8F). Similarly, for *Cyp11a1*, SREBP2 (57,922,776–57,922,785 bp) binding motifs were found within the gene but downstream of the predicted TSS (57,921,954 bp) (Figure 8G). These studies suggested that SREBP2 probably acts as an enhancer for these genes. In the *Hmgcr* gene we found SREBP2 binding motif upstream of the TSS in the promoter within a more accessible peak in the KO cells than the WT cells (Figure S4C). In *the Ldlr promoter, we found the* SREBP2 binding motif (21,634,861–21,634,870 bp) proximal to the TSS (Figure S5D). Therefore, MTP deficiency appears to enhance the chromatin accessibility of SREBP2 to augment the expression of key enzymes in cholesterol and steroid biosynthesis.

In summary, this study highlights the importance of MTP in the regulation of steroidogenesis and indicates that MTP deficiency has broad effects on the transcriptional landscape in MA-10 cells. MTP deficiency leads to increased expression and activation of SREBP2. In addition, MTP enhances the accessibility of SREBP2 to binding motifs in various promoters, thus potentially facilitating the upregulation of both the cholesterol and steroid biosynthesis pathways.

## Discussion

In this study, we describe the regulatory role of MTP in cholesterol and steroid biogenesis in Leydig cells. Biochemical and molecular studies identified significant upregulation of several genes in the cellular uptake, biosynthesis, and intracellular trafficking of cholesterol in MTP-deficient cells. In addition, we observed significant increases in the expression of genes in the conversion of cholesterol to progesterone in these cells versus WT cells. Furthermore, signaling pathways in steroid biosynthesis were upregulated in KO cells. Mechanistic studies suggested that MTP deficiency significantly enhanced the expression and activity of SREBP2. Therefore, our studies suggested that MTP restricts the expression and function of SREBP2 in regulating cholesterol and steroid biosynthesis in Leydig cells.

Evidence of the functional role of MTP in MA-10 cells was demonstrated through inhibition and KD studies (Figure 2). Both treatments significantly increased steroid secretion in Leydig cells (Figures 2A–2E). To gain a detailed understanding of MTP’s function in these cells, we obtained MTP KO cell lines. MTP-deficient MA-10 cells also secreted significantly more progesterone than WT control cells (Figure 3D). These studies indicated that MTP is a negative regulator of steroid production in Leydig cells.

MTP deficiency enhanced the expression of proteins involved in lipoprotein uptake and enzymes in cholesterol biosynthesis. Steroidogenic cells take up both LDL and HDL via Ldlr and SR-B1, respectively.^49^ Furthermore, the expression of several key genes in cholesterol biosynthesis increased, including *Hmgcr*, *Hmgcs2*, and *Insig1* (Figure 3H). Cholesterol is transported from the ER to the OMM by Stard4,^50^ and Aster B has been shown to transfer cholesterol from the ER to the mitochondria through contact sites in steroidogenic cells.^39^ StAR protein then transfers cholesterol from the OMM to IMM. Whereas StAR expression was unaltered, Stard4 and Aster B expression were significantly enhanced in MTP-deficient cells (Figure 3H), thereby indicating elevated cholesterol transfer to mitochondria. All these are targets of SREBP2, thus suggesting enhanced SREBP2 activity in the KO cells. Indeed, we observed increased expression and activity of SREBP2 in KO cells. ATAC-Seq demonstrated increased chromatin accessibility around SREBP2 motifs in target genes. The role of SREBP2 in the MTP-mediated regulation of cholesterol biosynthesis was confirmed through KD studies (Figures 6G–6I). Therefore, MTP appears to regulate several genes involved in cholesterol procurement, production, and delivery to mitochondria by restricting SREBP2 accessibility to various targets. Whether higher expression of SREBP2 itself might enhance chromatin accessibility, or other factors promote this accessibility in KO cells, remains to be determined.

Beyond cholesterol metabolism, MTP deficiency significantly affected steroid biosynthesis. MTP deficiency increased the mRNA and protein levels of enzymes in the biosynthesis of steroid hormones. To address whether the activity of enzymes in steroid biogenesis was enhanced, we provided specific cell-permeable substrates for P450scc and 3β-Hsd1 enzymes. Despite enhanced cholesterol uptake, biosynthesis, and trafficking, the provision of these substrates further enhanced steroidogenesis and consequently indicated increased activity of these enzymes (Figures 4D and 4E). Therefore, MTP deficiency enhances the expression and activity of enzymes in progesterone synthesis.

Leydig cells are responsive to LH, which interacts with Lhcgr on the cell surface and triggers the activation of multiple signaling pathways that enhance sex hormone production. Lhcgr activates the MAP kinase pathway by activating ras-raf kinases, which in turn activate Erk1/2 by phosphorylation.^51^ Additionally, Lhcgr activates adenylate cyclase, which increases intracellular cAMP concentration, thereby activating PKA.^51^ All these kinases further phosphorylate CREB and consequently activate several TFs in cells through phosphorylation. We observed that MTP deficiency enhanced Lhcgr expression (Figures 5D–5F). Furthermore, phosphorylation and activation of ERK1/2 and PKA were elevated in the KO cells (Figure 5G). Therefore, MTP deficiency also enhances LH signaling, which controls steroid production.

During steroidogenesis, stored triglycerides are hydrolyzed, and fatty acids are oxidized for energy. Unlike PLCs, MA-10 cells heavily rely on increased glycolytic rates to support steroidogenesis. We observed greater lipolysis and glycolysis rates in KO cells than in WT cells. In summary, MTP deficiency significantly influenced several facets of steroid biosynthesis, including hormone signaling and mitochondrial activity.

Unexpectedly, we observed increased expression and activity of SREBP2. The finding that knockdown of *Srebf2* reduced progesterone secretion in MTP KO cells suggested that increased SREBP2 activity enhanced steroidogenesis in these cells (Figure 6F). ATAC-seq showed increased accessibility of SREBP2 motifs in *Cyp11a1* and *Hsd3b1* genes in KO cells (Figures 8E and 8F). Therefore, elevated SREBP2 might directly interact with promoter enhancer sequences and consequently increase the transcription of these steroidogenic genes.

SREBP2 usually functions as a sensor of free cholesterol in cells. Cholesterol insufficiency in the ER in hepatocytes and fibroblasts increases SREBP2 activity and consequently the expression of several genes in cholesterol metabolism. Therefore, we anticipated that MTP deficiency might reduce ER cholesterol levels; however, biochemical measurements did not show significant reductions in ER cholesterol levels (Figure S1D). Therefore, MTP is likely to regulate SREBP2 expression through unknown mechanisms independent of cellular cholesterol levels. Lipids other than cholesterol might potentially regulate SREBP2 activity in Leydig cells. MTP has been suggested to bind various types of lipids by recognizing hydrophobic patches.^3^

The regulation of SREBP2 by MTP appears to be unique to Leydig cells. Transcriptomic analysis of MTP-deficient human hepatoma Huh7 cells revealed no change in SREBP2 expression in KO and WT cells.^52^ A similar cell-specific function of MTP in adipocytes has been described. MTP inhibits ATGL activity in adipocytes but not hepatocytes.^6^

To determine whether MTP regulates steroidogenesis exclusively by regulating SREBP2, we inhibited MTP in *Srebf2*-deficient cells. MTP inhibition increased progesterone secretion in Srebf2-deficient cells (Figure S4H), indicating that MTP also enhances progesterone secretion via SREBP2-independent mechanisms. Support for this conclusion is also obtained from ATC Seq studies. Besides the accessibility of SREBP2 to its target genes, ATAC sequencing showed that MTP deficiency also altered the accessibility of CEBP, USF2, SP1, and NR2F2 (Figure 8A). These studies indicate that MTP deficiency might affect the activity of several transcription factors.

In conclusion, our studies showed that sex hormone production is regulated by MTP in Leydig cells. MTP deficiency has a wide range of effects on the uptake, synthesis, and intracellular trafficking of cholesterol. In addition, it enhances hormone signaling pathways that regulate steroid production and enzymes involved in steroidogenesis. MTP might orchestrate most, but not all, of these diverse effects by regulating SREBP2 expression. Future studies might identify other TFs and mechanisms regulated by MTP that are involved in steroidogenesis and determine the importance of this regulation *in vivo*.

### Limitations of the study

Despite the extensive molecular, biochemical, and genomic approaches used in this study, several limitations should be acknowledged. First, most mechanistic experiments were performed in MA-10 cells, an immortalized mouse Leydig cell line. Although we obtained preliminary data in PLCs, validation in *in vivo* models will be essential to confirm their physiological relevance. Second, while our data demonstrate that MTP deficiency enhances SREBP2 expression, activity, and chromatin accessibility, the precise molecular mechanism by which MTP regulates SREBP2 remains unresolved. Notably, ER cholesterol levels were not significantly altered in MTP-deficient cells, suggesting that MTP may regulate SREBP2 through cholesterol-independent pathways, potentially involving other lipid species or ER lipid-sensing mechanisms. Recently, MTP has been detected in the nucleus.^53^ It remains to be determined whether nuclear MTP plays a role in chromatin structure and thereby alters the accessibility of different TFs to their target genes.

## Resource availability

### Lead contact

Further information and requests for resources and reagents should be directed to and will be fulfilled by the lead contact, Mahmood M. Hussain (mahmood.hussain@nyulangone.org).

### Materials availability

This study did not generate new unique reagents. Human hepatoma Huh7 cells (ABM, Catalog #T8973) and MA-10 cells (ATCC, Catalog #CRL-3050) were obtained from commercial sources. Mouse tissues were collected from 3 to 4-month-old male C57BL/6J mice obtained from The Jackson Laboratory (Strain #000664). There are no restrictions on the availability of materials used in this study. Details of all materials are provided in the key resources table, and primer sequences are listed in Table S1. Additional information is available from the lead contact upon reasonable request.

### Data and code availability

• The datasets generated during this study have been deposited in the NCBI Gene Expression Omnibus (GEO) and are publicly available. Bulk RNA-seq data are available under accession number GSE305422, and bulk ATAC-seq data are available under accession number GSE305421. These accession numbers are also listed in the key resources table. All other data supporting the findings of this study are available within the paper.
• This study did not generate new code. All analyses were performed using publicly available software and packages provided in the key resources table.
• Bulk RNA-seq and ATAC-seq data processing were performed by Novogene (Sacramento, CA). Any additional information required to reanalyze the data reported in this paper is available from the corresponding author upon reasonable request.

## Acknowledgments

We thank Drs. Michael Garabedian and Susan Logan for immunostaining testes and liver tissue in Figure 1A. We acknowledge Dr. Sujith Rajan for providing the NBD-TG vesicles for HSL assay, and his help with the operation of Sea Horse (Agilent) and Resipher (LucidLab) instruments. This work was supported by 10.13039/100000002National Institutes of Health (HL158054, HL166418, HL160470, and HL166214) grants to MMH.

## Author contributions

A.C.: conceptualization, data curation, formal analysis, investigation, methodology, and writing: original, review. M.B.T.: data curation, analysis, and writing: original, review. T.P.: data curation. B.G.: data curation. R.R.: data curation, Q.M.: discussion and writing: review. M.M.H.: conceptualization, supervision, project administration, resources, and writing - original, review, editing.

## Declaration of interests

Authors declare no competing interest.

## STAR★Methods

### Key resources table

**Table:** 

REAGENT or RESOURCE	SOURCE	IDENTIFIER
**Antibodies**		

Anti-MTP	BD Biosciences	Cat# 612022; RRID: AB_399306
Anti-MTP (IHC)	Abcam	Cat# ab63467; RRID: N/A
Anti-ApoB (IHC)	Abcam	Cat# ab20737; RRID: AB_726493
Anti-MTP (Gold-EM)	Thermo Fisher Scientific	Cat# PA5-102474; RRID: N/A
Anti-StAR	Cell Signaling Technology	Cat# 8449S; RRID: AB_10829092
Anti-Cyp11a1	Cell Signaling Technology	Cat# 14217S; RRID: AB_2798446
Anti-Hsd3B1	Santa Cruz Biotechnology	Cat# sc-515120; RRID: N/A
Anti-Lhcgr	Proteintech	Cat# 19968-1-AP; RRID: N/A
Anti-OxPhos cocktail	Abcam	Cat# ab110413; RRID: AB_2629281
Anti-CREB	Cell Signaling Technology	Cat# 9197S; RRID: AB_2561044
Anti-phospho-CREB	Cell Signaling Technology	Cat# 9198S; RRID: AB_2561045
Anti-PKA-C	Cell Signaling Technology	Cat# 4782S; RRID: N/A
Anti-phospho-PKA-C	Cell Signaling Technology	Cat# 5661S; RRID: N/A
Anti-ERK1/2 (p44/42)	Cell Signaling Technology	Cat# 9102S; RRID: AB_330744
Anti-phospho-ERK1/2	Cell Signaling Technology	Cat# 9101S; RRID: AB_331646
Anti-SREBP2	Abcam	Cat# ab30682; RRID: AB_778139
Anti-SF-1	Santa Cruz Biotechnology	Cat# sc-393592; RRID: N/A

**Chemicals, peptides, and recombinant proteins**		

8-Bromo-cAMP	Fisher Scientific	Cat# 1140/50
22(R)-Hydroxycholesterol	Sigma-Aldrich	Cat# H9384
Pregnenolone	Sigma-Aldrich	Cat# P9129-1G
Human chorionic gonadotropin (hCG)	Fisher Scientific	Cat# 23-073
Lovastatin	Fisher Scientific	Cat# 15-301-0
Lomitapide	Sigma-Aldrich	Cat# SML1385
HSL inhibitor	Santa Cruz Biotechnology	Cat# sc-206328
BODIPY 480/508-cholesterol	Cayman Chemical	Cat# 24618

**Critical commercial assays**		

Amplex Red Cholesterol Assay Kit	Thermo Fisher Scientific	Cat# A12216
CellTiter 96® AQueous One Solution	Promega	Cat# G3582
Dual-Glo® Luciferase Assay System	Promega	Cat# E2920
Progesterone ELISA Kit	Cayman Chemical	Cat# 582601
Testosterone ELISA Kit	Cayman Chemical	Cat# 582701
Pierce BCA Protein Assay Kit	Thermo Fisher Scientific	Cat# 23225

**Deposited data**		

RNA-seq	This study	GEO: GSE305422
ATAC-seq	This study	GEO: GSE305421

**Experimental models: cell lines**		

MA-10 mouse Leydig tumor cells	ATCC	Cat# CRL-3050; RRID CVCL_3891
Huh7 human hepatoma cells	Applied Biological Materials	Cat# T8973; RRID: CVCL_0336

**Experimental models: organisms/strains**		

Mice: C57BL/6J	The Jackson Laboratory	Strain #000664; RRID: IMSR_JAX:000664
Primary mouse Leydig cells	This study	–

**Oligonucleotides**		

Mttp sgRNA1	Synthego	Cat# 138125217
Mttp sgRNA2	Synthego	Cat# 138125227
Srebf2 sgRNA1	Synthego	Cat# 82169762
Srebf2 sgRNA2	Synthego	Cat# 82169781
qPCR primers	This study	Table S1

**Recombinant DNA**		

pSynSRE-T-Luc	Addgene	Cat #60444
pRL-TK	Promega	Cat# E2241

**Software and algorithms**		

GraphPad Prism	GraphPad Software	https://www.graphpad.com
ImageJ/Fiji	NIH	https://imagej.nih.gov/ij
BioRender	BioRender Inc.	https://biorender.com/
DESeq2 (v1.37.4)	DESeq2; Bioconductor	https://bioconductor.org/packages/DESeq2/
clusterProfiler (v4.5.1)	clusterProfiler; Bioconductor	https://bioconductor.org/packages/clusterProfiler/
dplyr (v1.1.4)	dplyr; tidyverse	https://cran.r-project.org/package=dplyr
Trim Galore (v0.6.7)	Trim Galore; Babraham Bioinformatics	https://www.bioinformatics.babraham.ac.uk/projects/trim_galore/
Bowtie2 (v2.4.2)	Bowtie2; Johns Hopkins University	http://bowtie-bio.sourceforge.net/bowtie2/index.shtml
Samtools (v1.22.1)	Samtools; Wellcome Sanger Institute	http://www.htslib.org
Picard MarkDuplicates	Picard; Broad Institute	https://broadinstitute.github.io/picard/
MACS2 (v2.2.7.1)	MACS2; Zhang Lab	https://github.com/macs3-project/MACS
Bedtools (v2.31.0) multicov	Bedtools; Aaron Quinlan Lab	https://bedtools.readthedocs.io/en/latest/
ChIPseeker (v4.1.1)	ChIPseeker; Bioconductor	https://bioconductor.org/packages/ChIPseeker/
HOMER (v4.11)	HOMER; Heinz Lab	http://homer.ucsd.edu/homer/
findMotifsGenome.pl	findMotifsGenome.pl; Heinz Lab	http://homer.ucsd.edu/homer

**Other**		

DMEM/F12 medium	Fisher Scientific	Cat# 11-330-032
Horse serum	Thermo Fisher Scientific	Cat# 16050122
Collagenase IV	Gibco	Cat# 17104-019
Endofectin Max transfection reagent	Genecopoeia	Cat# EF013
TRIzol reagent	Thermo Fisher Scientific	Cat# 15596018
RIPA buffer	Thermo Fisher Scientific	Cat# J63306.AP
Protease inhibitor cocktail	Sigma-Aldrich	Cat# P2714
Phosphatase inhibitor cocktail	Sigma-Aldrich	Cat# P0044
Nitrocellulose membrane	Amersham	Cat# 10600002
Gelatin	Merck	Cat# G1393
Neon Transfection System Kit	Thermo Fisher Scientific	Cat# MPK1096
Protein A/G PLUS Agarose	Santa Cruz Biotechnology	Cat# sc-2003

### Experimental model and study participant details

#### Animals

Male C57BL/6j mice (JAX: #000664) were purchased from the Jackson Laboratory. All experiments used male mice, aged 12-16 weeks. All animals were maintained in the Animal Core Facility under a 12 h light/dark cycle with free access to food (chow diet, PicoLab® Catalog #5053) and water. All animal procedures were approved by the Institutional Animal Care and Use Committee of New York University Langone Health (protocol #201900080) and conducted in accordance with institutional and NIH guidelines. All primary cell isolations were performed using male mice, consistent with the focus on Leydig cell steroidogenesis. Therefore, sex-based differences were not evaluated *in vivo*, which represents a limitation of the study. Age, genotype, and housing conditions were consistent across all animals unless otherwise noted.

#### Cell lines and culture conditions

Huh7 cells were cultured as previously described.^54^ MA-10 mouse Leydig tumor cells were maintained in DMEM/F12 medium supplemented with 15% horse serum and antibiotics in a humidified incubator at 37°C with 5% CO_2_. Cells were routinely passaged for no more than 5–6 passages per experiment. For experiments, cells were seeded on 0.1% gelatin-coated six-well plates at a density of 3 × 10^5^ cells per well and allowed to adhere for 48 h prior to treatment. Cells were stimulated with 0.5 mM 8-bromo cyclic AMP (8-BrcAMP) or 20 ng/mL hCG for 3 h to induce steroidogenesis. For enzymatic activity assays, cells were incubated with 10 μg/mL 22(R)-hydroxycholesterol or 1 μg/mL pregnenolone for 3 h in the presence or absence of 8-BrcAMP. For cholesterol synthesis inhibition, cells were treated with 0.5 μM lovastatin for 16 h in serum-free F12 medium, followed by stimulation with 8-BrcAMP for 3 h. Conditioned media were collected for progesterone quantification by ELISA.

All cell lines were routinely monitored for morphological consistency by phase-contrast microscopy at each passage, including assessment of characteristic morphology, adherence, growth rate, and absence of contamination. Functional validation of Leydig cell steroidogenic activity, including responsiveness to 8-BrcAMP or hCG stimulation and hormone production, was periodically performed to confirm phenotypic stability of the cell models. Cell lines were obtained from authenticated commercial sources as described in the key resources table. All cell cultures were routinely tested for contamination prior to experimentation.

### Method details

#### Immunostaining and in situ hybridization

Human liver and testis slides were obtained from Novus Biologics and stained for MTP (1:500 dilution) and ApoB (1:500) with polyclonal antibodies. Goat anti-rabbit IgG (Vector Lab) was used as the secondary antibody. Images were taken at 20× magnification. Paraffin fixed sections of mouse tissues (4 μm) from 3-month-old male mice were used for *in situ* hybridization with RNAScope following the manufacturer’s protocol. The BA-Mm-Mttp-3zz-St-C1 (ACD, 1217461-C1) probe used for hybridization was designed and synthesized by Advanced Cell Diagnostics. Slides were stained with H & E (liver) or DAPI (testis) and mounted with ProLong Gold antifade reagent for visualization at a 561 nm wavelength with a Nikon Eclipse Ti confocal microscope at 40× magnification.

#### Determination of MTP activity in MA10 cell homogenates and mouse testis tissue

At 48 h after plating, confluent MA10 cell monolayers were washed with cold phosphate buffered saline (PBS) and collected in 250 μL of buffer K (1 mM Tris, 1 mM EGTA, and 1 mM MgCl2, pH 7.6) supplemented with protease inhibitor cocktail (PIC, 10 μL/mL). For homogenization, cell suspensions were sonicated with two pulses at 40% frequency for 10 s, with a 5 s rest between pulses, then centrifuged at 13,000 g for 15 minutes. Frozen mouse testis tissues were homogenized in 1 mL buffer K and 0.1% NP-40 with PIC via a Dounce homogenizer, sonicated, and centrifuged. Supernatants were used for experiments. The protein concentrations in the homogenates were estimated with the bicinchoninic acid (BCA) method with various concentrations of bovine serum albumin as the standard. For determination of the triglyceride transfer activity of MTP, various amounts of homogenates were incubated with donor vesicles containing NBD-labelled triolein and acceptor vesicles. Fluorescence was measured at different time periods at 460 nm excitation and 530 nm emission. The percentage TG transfer was calculated.^55^

#### MTP inhibition in MA10 cells

MA10 cells were seeded in six-well plates at a density of 300,000 cells. After 48 h, the medium was replenished containing either DMSO or 1 μM lomitapide for 16 h. Cells were washed and treated with 0.5 mM 8-BrcAMP for 3 h in serum-deprived F12 medium. Subsequently, the culture medium was collected for progesterone secretion measurement by ELISA.

#### Electroporation with sgRNA-cas9

We selected sgRNA for both Mttp (#138125227: GCCGCUCAUUAUUUAAUGAG; #138125217: GCCUCUCAUUAAAUAAUGAG) and Srebf2 (#82169762: ACAGUUUGUCAGCAAUCAAG; #82169781: GUUGCUCUGAAAACAAAUCA) genes with the Synthego CRISPR design tool to minimize off target effects. Electroporation was performed in MA10 cells with a Neon transfection system (Thermo Scientific, MPK5000) with a 1:3 molar ratio of Cas9 and sgRNA, and 100,000 cells each time.^54^ Briefly, Spcas9 2NLS nuclease (30 pmol) was incubated with 2 sgRNA (90 pmol) for 15 min at room temperature. About 80% confluent MA10 cells were treated with trypsin, washed twice with PBS, and resuspended in R buffer (Neon transfection system 10 μL kit). Cells (100,000 in 5 μL) were mixed with 7 μL RNP mixture and exposed to two pulses of 1350 V for 20 milliseconds.

#### Clonal selection

Transfected MA10 cells grown to 80% confluence were serially diluted and seeded in a 96-well plate to distribute one cell per well. The cells were cultured for several days to allow for microcolony formation. Microcolonies were subsequently expanded and screened for MTP knockout (KO).^54^

#### Steroid measurement by ELISA

Cells were cultured for 48-72 h and stimulated with or without 0.5 mM 8-BrcAMP for 3 h in serum-free F12 medium. The medium was collected for steroid quantification with progesterone/testosteroneELISA kit (Cayman) according to the manufacturer’s protocol. The kits provide a detection range of 7.8-1000 pg/mL (progesterone) and 3.9-500 pg/ml (testosterone) with the sensitivity of detection approximately 10 pg/mL; hence all the samples were serially diluted thousand-fold with media before assay. The amounts of steroid secreted were normalized to total cell protein.

#### qRT-PCR

Total RNA from MA10 cells was isolated with TRIzol reagent according to the manufacturer’s protocol. RNA quality and quantity were assessed with Nanodrop spectrophotometer (Thermo Scientific, ND-ONE-W). The cDNA was synthesized with random primers and 2 μg total RNA with High-Capacity cDNA Reverse Transcription Kit according to the manufacturer’s protocol. The qPCR was performed with appropriately diluted cDNA and RNA controls with 2× qPCR Mix (Accuris) in Real time PCR QuantStudio3 (Applied Biosystems, A28131). Gene expression was calculated as fold change with the ddCt method, with the Ct value of 18S rRNA serving as a reference control. Primer sequences are in Table S1.

#### Western blotting

MA10 cells were harvested in 1× RIPA buffer containing 1% (v/v) PIC, then centrifuged at 13,000 g for 15 mins at 4°C. Protein concentrations were estimated with bicinchoninic acid assays. For detection of unphosphorylated/phosphorylated proteins, cells were stimulated with 8-BrcAMP for 20 minutes and harvested in buffer K containing 0.1% NP-40, and protease and phosphatase inhibitor cocktails. Equal amounts of lysates were loaded on 12% SDS-PAGE gels and transferred to nitrocellulose membranes after electrophoresis. The membranes were then incubated with antigen-specific primary antibodies overnight at 4°C (See key resources table). After several washes, the membranes were incubated with secondary antibodies for 1 h at room temperature and developed with a chemiluminescence kit. Protein bands were quantified in Image J software (NIH).

#### Immunoprecipitation

For detection of MTP protein in mouse testis and MA10 cells, ∼400–500 μg protein lysates were precleared with 2 μL goat anti-mouse IgG (Invitrogen) and incubated with anti-MTP antibody (BD Bioscience) overnight at 4°C. The protein-antibody complexes were precipitated with agarose beads, and the protein was eluted with 4× Laemmli buffer.

#### Immunogold labeling and electron microscopy

Monolayers of MA10 cells in 60 mm culture dishes were treated with 8-BrcAMP for 3 h and washed three times. Cells were fixed in 4% paraformaldehyde/1% glutaraldehyde in 0.1 M sodium cacodylate buffer, pH 7.2, dehydrated in a graded ethanol series, embedded in LR White resin, and cured at 55°C. The blocks were sectioned with a Reichert Ultracut microtome at 70 nm and picked up on formvar coated nickel grids. The resulting grids were then floated on 10 mM sodium citrate at 99°C for 30 min to expose antigenic sites, permeabilized in 0.25% Triton X-100 in PBS, blocked with 5% goat serum in PBS with 0.01 M glycine, rinsed in TBS, and exposed to rabbit polyclonal MTP antibody in TBS with 1% bovine serum albumin overnight at 4°C. The next day, the grids were exposed to a 1:20 dilution of goat anti-rabbit IgG conjugated with 10 nm gold (Electron Microscopy Sciences, Hatfield, PA) in PBS with 0.01 M glycine for 1 h, rinsed in PBS, and fixed in 2% glutaraldehyde solution in PBS. The grids were then rinsed in distilled water, dried, and visualized with a Zeiss EM 900 transmission electron microscope retrofitted with an SIA L3C digital camera (SIA, Duluth, GA).

#### Primary leydig cell isolation

Primary Leydig cells were isolated from 4-month-old male C57BL/6J mice using a collagenase digestion method as previously described.^56^ Briefly, decapsulated testes were digested in collagenase type IV at 37°C for 10 min with gentle agitation. Interstitial cells were separated from seminiferous tubules by sequential filtration through 100 μm and 40 μm cell strainers, followed by red blood cell lysis using ACK lysis buffer. Cells were seeded in DMEM/F12 medium supplemented with 15% horse serum and penicillin/streptomycin. Myeloid cells were removed by hypotonic KCl treatment after 24 h. Cells were cultured for an additional 48 h prior to experiments. Cell purity was assessed by qPCR analysis of Leydig cell marker *Cyp11a1*, Sertoli cell marker *Rhox5*, and spermatogonia marker *Dbil5*.

#### Subcellular organelle separation and quantification of free cholesterol

Cells were seeded in 150 mm dishes, stimulated with 8-BrcAMP for 3 h, and harvested for subcellular fractionation.^57^ Briefly, cells were scraped from culture dishes in 4 mL microsome isolation stability buffer containing sucrose and KCl, then centrifuged. Subsequently, cell pellets were resuspended in swelling buffer, homogenized with a Dounce homogenizer (25–30 strokes), and sonicated (25% frequency) with 2-second pulses for two to three times. The homogenized suspension was then subjected to a series of centrifugation steps to finally separate ER.^57^ The isolated ER fractions were resuspended in RIPA buffer containing PIC, and purity was assessed with western blotting.

#### Cholesterol quantification

Cells (3×10^5^) were plated in six-well plates and incubated in growth medium for 48 h. Lipids were extracted from cells by incubation of cell monolayers in 1 mL of 100% isopropanol overnight at 4°C. The isopropanol from each well was collected in microcentrifuge tubes and dried, and lipids were resuspended in 100 μL isopropanol. For quantification of cholesterol from total cell lysates, 10 μL of each sample was added to a 96-well plate. Subsequently, 90 μL of cholesterol reagent (Pointe Scientific) was added and incubated at 37°C for 15 minutes. Absorbance was measured at 490 nm. Total and free cholesterol from ER fractions were quantified with the Amplex Red assay kit, which was selected for its high sensitivity.

#### Dual luciferase assays

MA10 cells were co-transfected with the pSyn-T-Luc and pRL-TK plasmids containing firefly luciferase (fused with the Hmgcs1 promoter containing an SREBP2 binding site) and Renilla luciferase (internal control), respectively. After 48 h, cells were incubated for 8 h in serum-free medium to activate SREBP2. Luciferase assays were performed 48 h post transfection according to the user manual. Both firefly and Renilla luminescence were recorded with a BioTek plate reader. Background measurements from non-transfected cells were subtracted, and the ratios of firefly to Renilla luminescence are reported.

#### Oxygen consumption rates in cells

For mitochondrial respiration activity studies, MA10 cells were seeded in 96 well plates compatible with the Lucid plate reader and incubated in a CO2 incubator at 37°C. After 16 h, the plate reader was mounted on the plate, and oxygen consumption rates (OCR) were recorded for 48 h in Resipher (LucidLab) and plotted after normalizing to total protein. For assessment of extracellular acidification rates, MA10 cells were seeded in 24 well culture plates (xFe extracellular flux analyzer) in F12 medium. After 16 h, the medium was replaced with assay medium (Agilent Technologies., Inc., catalog #103575-100) and cells were further incubated for 2 h at 37°C. Changes in oxygen consumption was measured with Seahorse (Agilent Technologies., Inc., catalog #103015-100) after adding 1 μM oligomycin, 1 μM FCCP, and 0.5 μM rotenone/antimycin A mixture according to manufacturer’s instructions. The resulting ECAR values were plotted after normalizing with total protein concentrations.

#### Free fatty acid quantification

Nitrobenzoxadiazole-labeled triacylglycerol [(NBD-TAG; 1,3-di(cis-9-octadecenoyl)-2-((6-(7-nitrobenz-2-oxa-1,3-diazol-4-yl)amino)hexanoyl)glycerol)] vesicles were prepared as described previously.^58^ WT and KO cell lysates (100 μL, 50 μg) were incubated with NBD labeled TG vesicles (10 μL, 3155 pmol) in the presence of DMSO or HSLi (20 μM) for 1 h. Free fatty acids were then extracted from the samples with 1 mL chloroform: methanol mixture (1:1 v/v) and 400 μL 0.1 M potassium carbonate. The samples were vortexed and then centrifuged (800 g for 10 min). The upper aqueous layer (200 μL) was collected, and fluorescence was measured at 460 nm excitation and 530 nm emission in a Perkin Elmer Inspire multi plate reader. The fluorescence values of blank samples were subtracted from the final measurements.

#### MTT assay for cell viability

MTT assays were performed with a CellTiter 96® Aqueous One Solution Cell Proliferation Assay kit according to the user manual. Briefly, 1,000 cells were plated in 96-well plates and maintained in growth medium at 37°C for different time periods. At the time of the assay, medium from each well was replaced with 100 μL fresh culture medium, and 20 μL of MTT reagent was added. Plates were incubated in a humidified chamber at 37°C for 3 h and the absorbance was recorded at 490 nm.

#### Bulk RNA-Seq and data analysis

Wildtype (WT, *n* = 4) and MTP KO (KO, *n* = 4) MA10 cells plated in triplicate were stimulated with 8-BrcAMP for 3 h, and total RNA was extracted. RNA integrity numbers (RIN) were computed to ensure high-quality RNA. Subsequently, mRNAs were isolated with polyU columns. cDNA libraries were generated and sequenced, and data were processed by Novogene (Sacramento, CA). Differential expression analysis between WT and KO cells (*n* = 4/group) was performed with DESeq2 (v.1.37.4) in the Bioconductor R package (v.4.2.1). The expression of genes with a p_adj_ value less than 0.05 (p < 0.05) and log2 fold change |0.5| were considered significant. Differentially expressed genes were visualized through volcano plots and heatmaps. Gene Ontology (GO) and KEGG pathway enrichment analysis of differentially expressed genes were conducted with clusterProfiler (v. 4.5.1) in the Bioconductor R package.^59^ KEGG and GO terms with cutoffs of p_adj_ <0.05 and log_2_ fold change |0.5| were considered significant. Differentially expressed TFs were retrieved for additional studies.^60^ Gene symbols from the normalized RNA-seq dataset were compared with the mouse TF gene list from AnimalTFDB with R (version 4.3.1) and the dplyr tool (version 1.1.4).

#### ATAC-seq and data analysis

WT and KO cells (*n* = 2 biological replicates per group) were treated with 8-BrcAMP for 3 hours under identical conditions to those for RNA-seq. Cell viability was assessed with Trypan blue assays. Nuclei were isolated and resuspended in the Tn5 transposase reaction mixture and incubated at 37°C for 30 min. Equimolar concentrations of Adapter 1 and Adapter 2 were added subsequently, and PCR was performed to amplify the library. Libraries were purified using AMPure beads, and the quality assessed by a Qubit. The index-coded samples were clustered on a cBot Cluster Generation System with a TruSeq PE Cluster Kit v3-cBot-HS (Illumina) according to the manufacturer’s instructions. After cluster generation, the libraries were sequenced on the Illumina HiSeq platform, and 150 bp paired-end reads were generated. All the above procedures from nuclei extraction onward were performed by Novogene (Sacramento, CA).

For analysis of the reads, raw FASTQ files were adapter-trimmed with Trim-galore (v0.6.7). Bowtie2 (v2.4.2) was used to align the reads to the mouse reference genome (mm39), with parameters optimized for short, paired reads. Alignments were filtered for adequately paired and uniquely mapped reads (MAPQ > 30). After removal of mitochondrial reads with samtools (v1.22.1),^61^ PCR duplicates were deleted with Picard MarkDuplicates. MACS2 (v2.2.7.1)^62^ was used to identify peaks. Peaks that overlapped with ENCODE blacklist regions were filtered out with Bedtools. Iterative overlapping was used to fuse replicate peaks thus resulting in a unified peak collection for downstream analysis. The read counts per merged peak were calculated with Bedtools (v.2.31.0) multicov. Normalization and differential peak analysis of WT and MTP KO MA10 cells were performed with DESeq2 (Bioconductor v1.37.4), and peak annotations were performed with ChIPseeker (v. 4.1.1).^63^ Differentially annotated regions were identified as peaks with |log_2_ fold change| >1 and adjusted p-value <0.05. Scatter plots of log_2_ fold changes in accessibility peaks (KO vs. WT) were created to identify acquired and lost peaks. Motif enrichments in differential peaks were performed with HOMER (v4.11)^64^ and findMotifsGenome.pl on the mm39 genome, with matching background sequences.

### Quantification and statistical analysis

Each experiment was performed at least three times. Biochemical measurements were made in triplicates. All graphs were plotted in Prism software (Version 9, GraphPad Software). Data are expressed as mean ± standard deviation (SD). Statistical significance was determined with Student’s t-test for two groups or one-way ANOVA followed by multiple comparisons for comparison of the means of more than two groups. The statistical significance represented by ∗, ∗∗, ∗∗∗, ∗∗∗∗ refer to *p* < 0.05, 0.01, 0.001, and 0.0001 respectively.
