# Supplementary material for: Microsomal triglyceride transfer protein restricts steroid production in Leydig cells by regulating SREBP2
Source: iScience. 2026 Apr 20;29(6):115817. doi: 10.1016/j.isci.2026.115817 (PMC13213752; doi:10.1016/j.isci.2026.115817)
Supplement: Document S1. Figures S1–S5, Table S1, and Data S1 [file mmc1.pdf]

iScience, Volume *29*

Supplemental information

Microsomal triglyceride transfer protein restricts steroid production in Leydig cells by regulating SREBP2

Atrayee Chattopadhyay, Munichandra Babu Tirumalasetty, Thomas Palaia, Bhargavi Gangula, Rachel Ruoff, Qing Robert Miao, and M. Mahmood Hussain

List of supplementary materials

- **Figures S1-S5**
- **Table S1**
- **Data S1. Full Western blots for the images presented in the article**

Supplementary figures

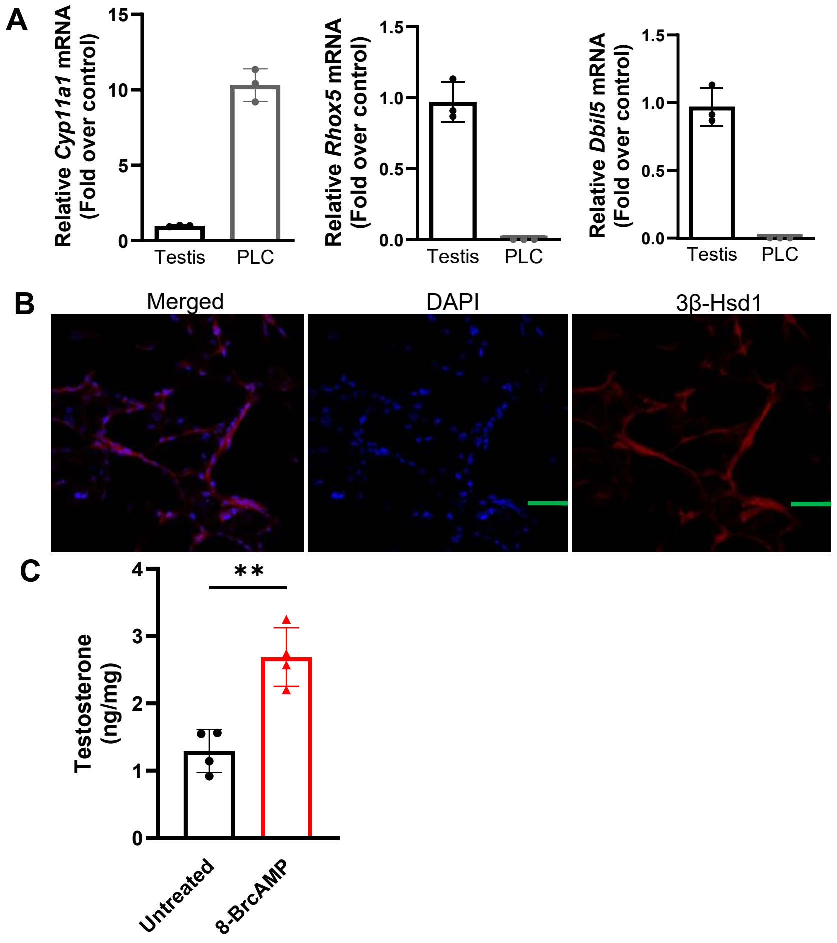

Figure S1, Related to Figure 2. Cultured mouse primary Leydig cells respond to 8-BrcAMP and increase testosterone secretion.

(A) Primary Leydig cells (PLC) were isolated and cultured from 4 month-old mice testes. After 48 h, mRNA levels of different marker genes were quantified and compared with their levels in whole testis. **(B)** Cells immune-stained with antibodies against 3β-Hsd1. Scale bar, 100 µm. **(C)** Primary Leydig cells from four-month-old mice were isolated and cultured for 72 h and stimulated with 8-BrcAMP for an additional 3 h in serum-free media. Testosterone was measured from the media and normalized with total protein. ***p* <0.01, unpaired *t*-test.

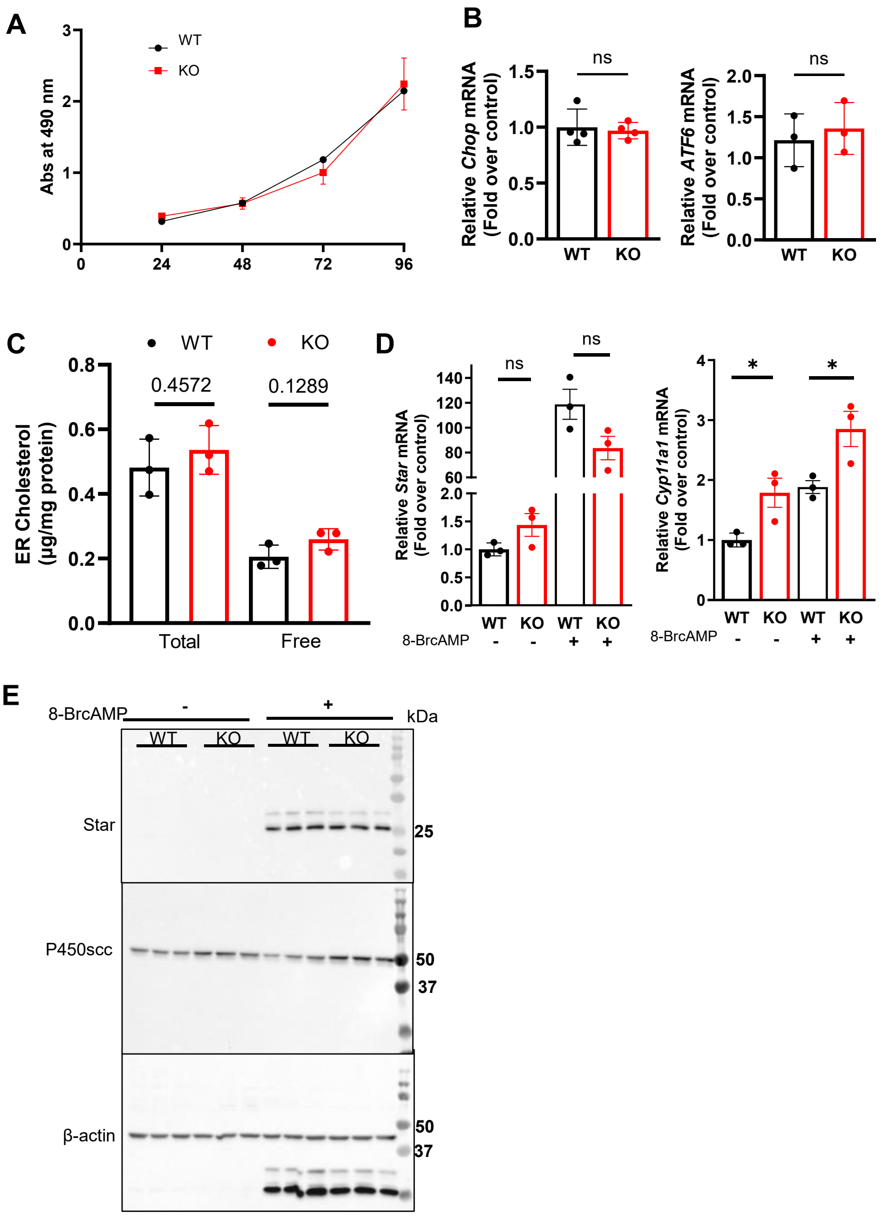

Figure S2, Related to Figures 3,4. MTP ablation has no effect on cell growth and expression of ER stress markers.

(A) Both WT and KO cells were seeded in 96 well plates to assess time-dependent cell growth. Representative of 3 independent experiments. (**B**) mRNA levels of ER stress marker genes CHOPS and ATF6 in the WT and KO MA-10 cells. Representative of 3 independent experiments. Mean ± SD (*n* = 3). (**C**) Total and free cholesterol levels in the ER of WT and KO cells. Representative of 3 independent experiments. Mean ± SD (*n* = 3). (**D**) mRNA levels of *Star* and *Cyp11a1* in the MTP KO clones after 8-BrcAMP stimulation. Mean ± SD (n = 3); ns, not significant, **p* <0.05, unpaired *t*-test. (**E**) Immunoblots showing Star and P450scc protein levels in 8-BrcAMP induced and uninduced cells.

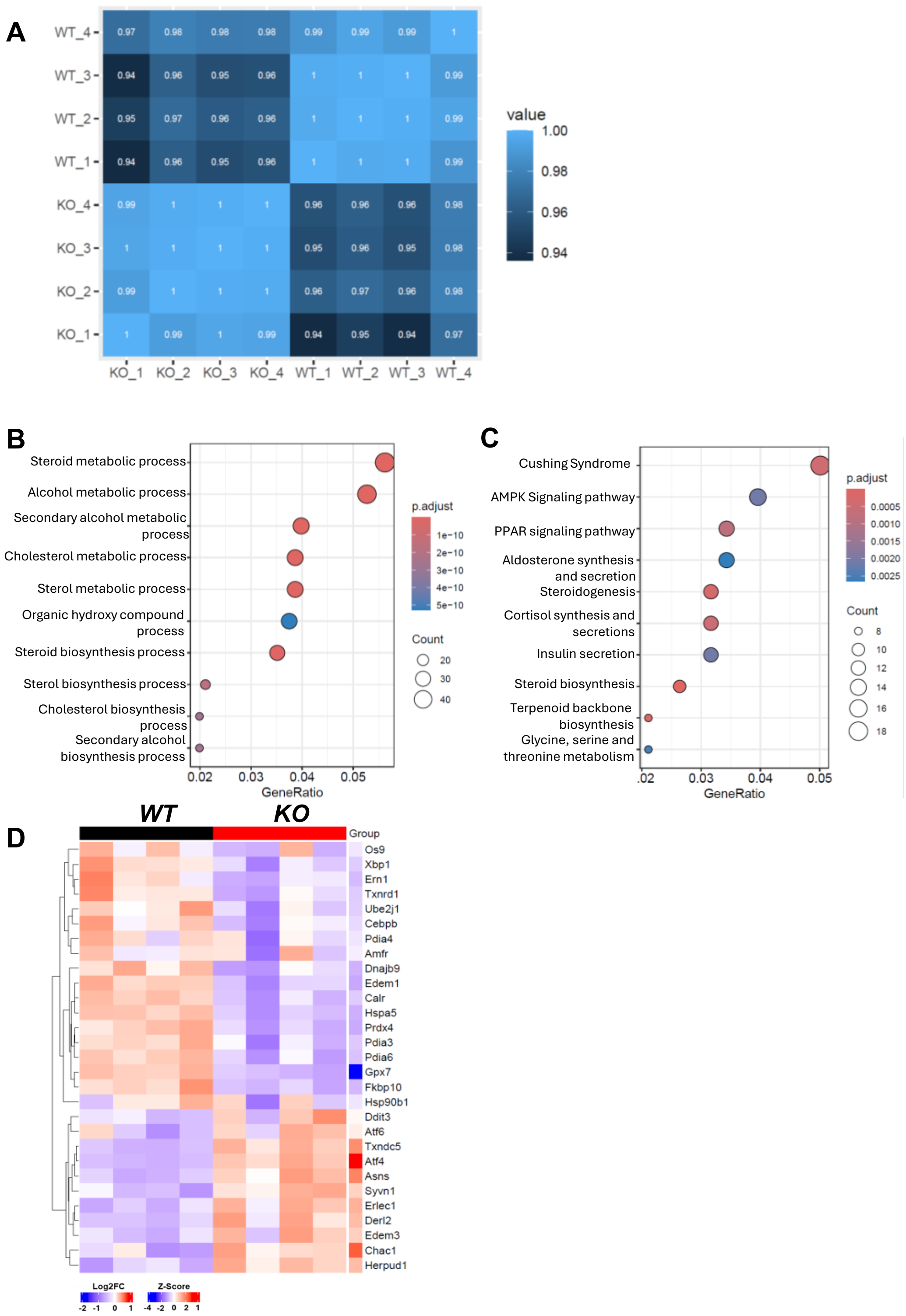

Figure S3, Related to Figure 5. Transcriptomic analysis and functional enrichment of different pathways in MTP KO cells

(A) Sample correlation heatmap showing high reproducibility among biological replicates of WT and KO groups based on RNA-seq gene expression profiles. **(B)** Top 10 upregulated Gene Ontology (GO) biological

process (BP) terms enriched in MTP KO cells compared to WT cells. Enrichment was calculated using clusterProfiler, with dot size indicating the number of genes per term and color representing adjusted p-value. Prominent processes include steroid metabolic process, cholesterol biosynthesis and steroid biosynthesis. **(C)** Top 10 upregulated KEGG pathways enriched in MTP KO cells compared to WT cells. Pathways include AMPK signaling, PPAR signaling, steroidogenesis, aldosterone and cortisol synthesis, and terpenoid backbone biosynthesis. Dot size reflects gene counts, and color scale indicates adjusted p-value. **(D)** Heat map of ER stress related genes from RNA seq data.

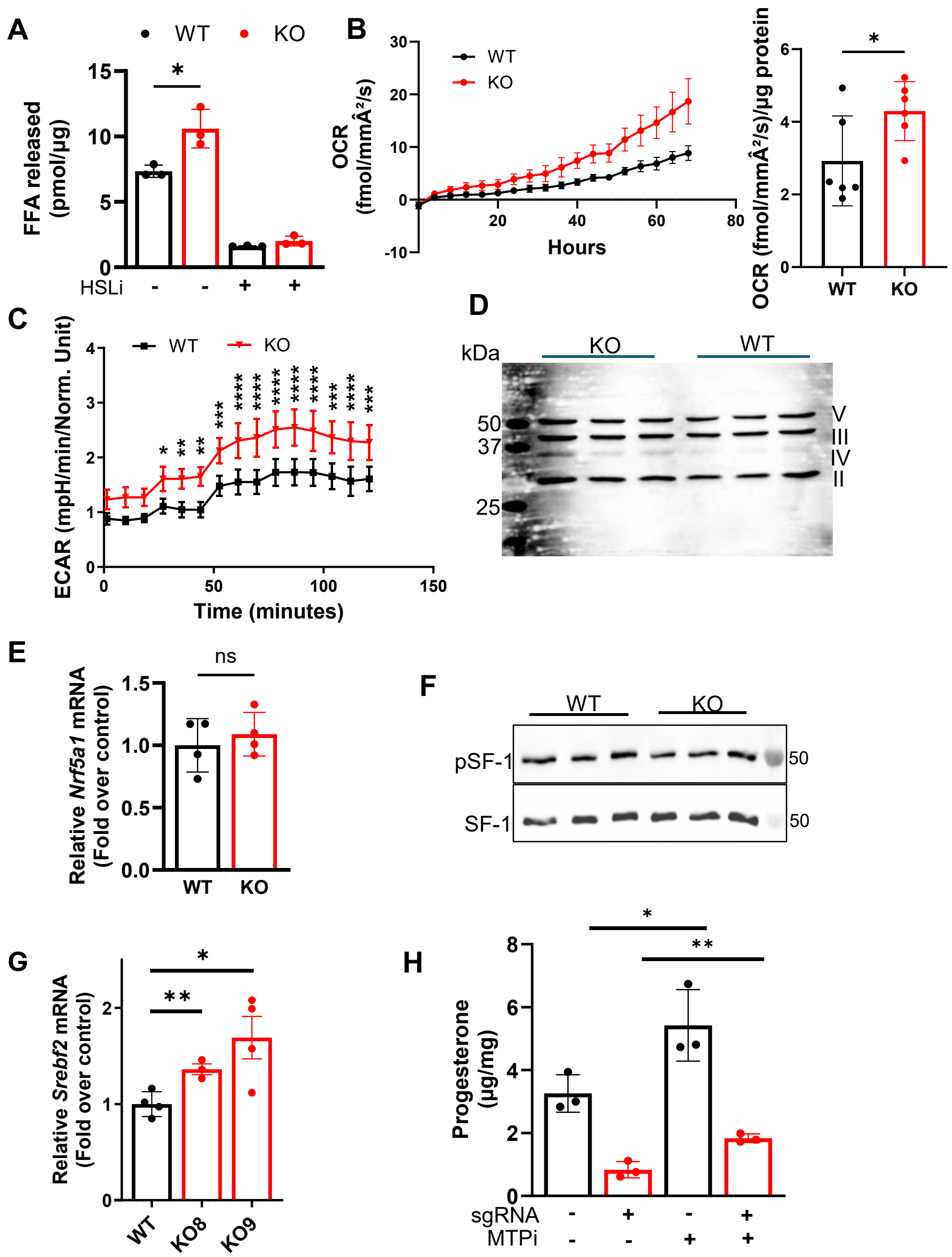

Figure S4, Related to Figures 5,6. MTP KO increases triglyceride hydrolysis and glycolysis rates in MA-10 cells

(A) Quantification of free fatty acids released from WT and KO cells after 8-BrcAMP stimulation treated without and with HSL inhibitor. Representative of three independent experiments. Mean ± SD (*n* = 3), * *p*<0.05, unpaired *t*-test. **(B)** Oxygen consumption rates (OCR) were measured in WT and KO cells using Resipher (left). The bar graph (right) shows OCR normalized to total protein. Representative of three independent experiments. Mean ± SD (*n* = 6), **p*<0.05, unpaired *t*-test. **(C)** ECAR measured in 8-BrcAMP-treated WT and KO cells normalized to total cell protein. Representative of three independent experiments. Mean ± SD (*n* = 5); *, **, ***, **** represent *p*<0.05, 0.01, 0.001, and 0.0001 respectively,

as determined by two-way ANOVA. **(D)** Western blot for mitochondrial complexes using ox-phos antibody in WT and KO cells. Representative of three independent experiments. **(E)** qPCR showing *Nr5a1* mRNA levels in both cell types after 8-BrcAMP stimulated conditions. Representative of three independent experiments. Mean ± SD (n = 4); ns, not significant, t-test. **(F)** Total and phosphorylated SF-1 protein levels in both cell types after 20 minutes of 8-BrcAMP stimulations. Representative of three independent experiments. (**G**) *Srebf2* mRNA levels in MTP KO clones KO8 and KO9 induced with 8-BrcAMP. Mean ± SD; *p<0.05, **p< 0.01, unpaired t-test. (**H**) Progesterone secretion in 48h cultured wildtype MA-10 cells under indicated conditions. Mean ± SD (n = 3); *p<0.05, **p< 0.01, unpaired t-test.

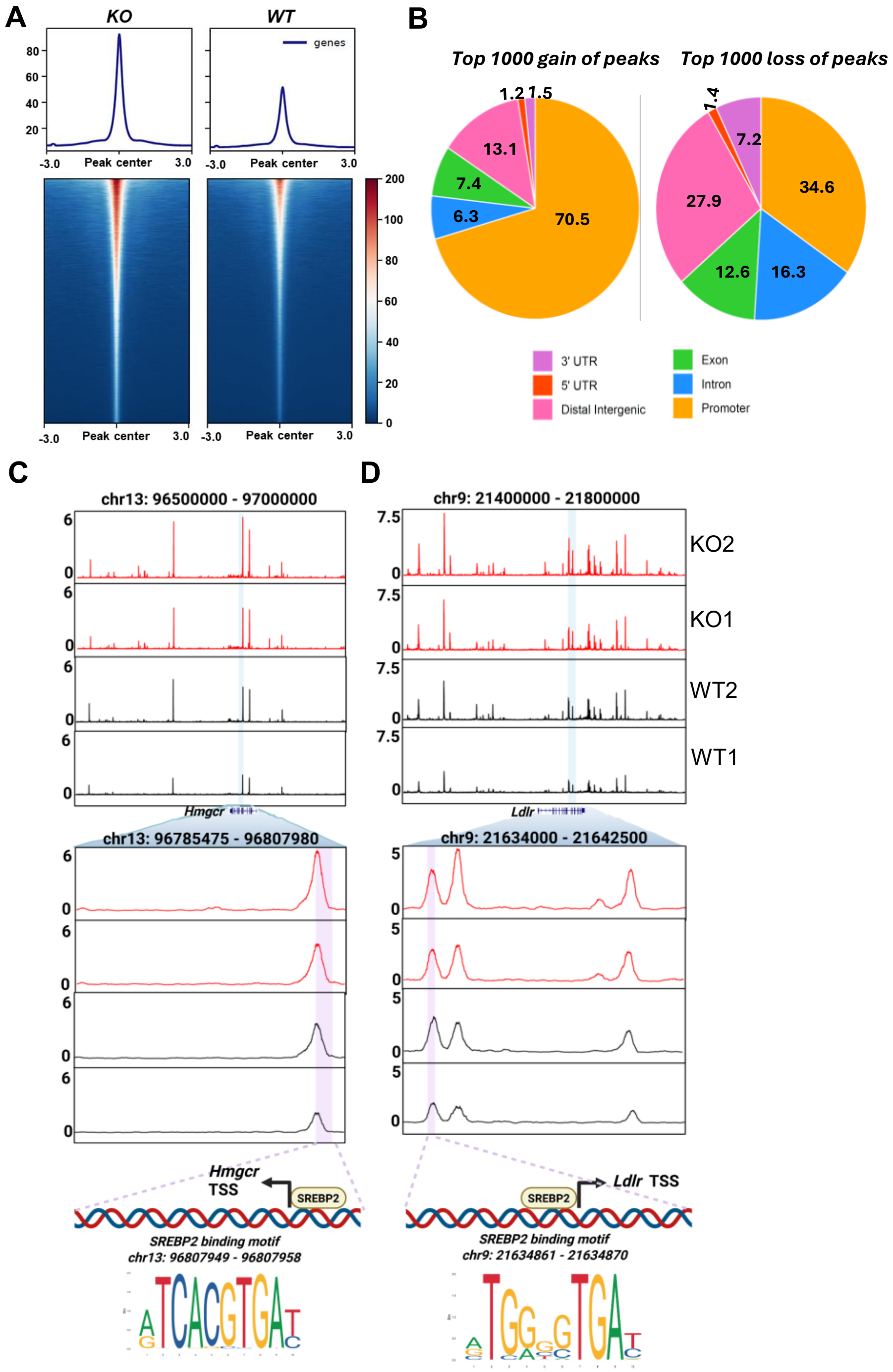

Figure S5, Related to Figure 7. Chromatin accessibility changes in WT and KO cells
(A) Aggregate accessibility profiles (top) and heatmaps (bottom) of all consensus peaks centered on peak centers (±3 kb). KO cells show more gain of accessibility signals at the peak centers, indicating higher signal intensity compared to WT cells. **(B)** Genomic locations of changes among the top 1000 gain (top) and loss of accessibility (bottom) peaks in MTP KO cells. **(C-D)** ATAC-Seq signals for the SREBP2 binding sites in the *Hmgcr* (C) and *Ldlr* (D) gene loci.

Table S1, Related to STAR Methods. Primers used for mRNA quantifications

Gene name	Forward sequence (5’-3’)	Reverse sequence (5’-3’)
Mttp	GACCACCCTGGATCTCCATA	AGCGTGGTGAAAGGGCTTAT
ApoB	TCCATATTCCAGACAACCTCTTC	GTTTATTTTGTTCCTGTTCATTGTGT
Insig1	GTGATAGCCACCATCTTCTCC	TACAGTAAACCGACAACAGCC
Hmgcr	CTTGTGGAATGCCTTGTGATT	AGCCGAAGCAGCACATGAT
Stard4	CCTGGCGCTTGGACTGGGAC	AGCTGCCCAGCAGTGGTGTAAC
Sqle	TGTTGCGGATGGACTCTTCTCC	GTTGACCAGAACAAGCTCCGCA
Mvd	ATGGCCTCAGAAAAGCCTCAG	TGGTCGTTTTTAGCTGGTCCT
Lss	CTCCAGAATGAGTTGGGTCGG	CGCTTTTGGTAAGTCCGTGAAA
Fdps	GGAGGTCCTAGAGTACAATGCC	AAGCCTGGAGCAGTTCTACAC
Cyp51	TGGAGCGAAAAGTCCACCAC	TGCATCACTCCCCAGAAGGTA
Abca1	GCTTGTTGGCCTCAGTTAGG	GTAGCTCAGGCGTACAGAGAT
Ldlr	TGACTCAGACGAACAAGGCTG	CTAACTAAACACCAGACAGAGGC
Scarb1	GCTGCGCTCGGCGTTGTCAT	GGGACGGGGATCTCCTTCCA
Srebf2	AGCAGGTGCAGACGGTACAG	CAGCGTGGTCAACACAAGGG
Gramd1b	TCCCAATGCCATCCAAGTC	ACAAAGTGCCAGAGCTCC
Star	AGTATTGACCTGAAGGGGTGGC	GGTGGTTGGCGAACTCTATCTG
Cyp11a1	AAAGACCGAATCGTCCTAAACC	CTTGATGCGTCTGTGTAAGACT
Hsd3b1	TGGACAAAGTATTCCGACCAGA	GGCACACTTGCTTGAACACAG
Cyp17a1	CCAGATGGTGACTCTAGGCCTCTTGTC	GGTCTGTATGGTAGTCAGTATCG
Lhcgr	CGCCCGACTATCTCTCACCTA	GACAGATTGAGGAGGTTGTCAAA
Nr5a1	AGCCAAAAGCCGCTCTGAC	CCACCTCCAGCTCCTTAAAGAC
18S	AGTCCCTGCCCTTTGTACACA	GATCCGAGGTCACTAAAC

Data S1. Full Western blots for the images presented in the article

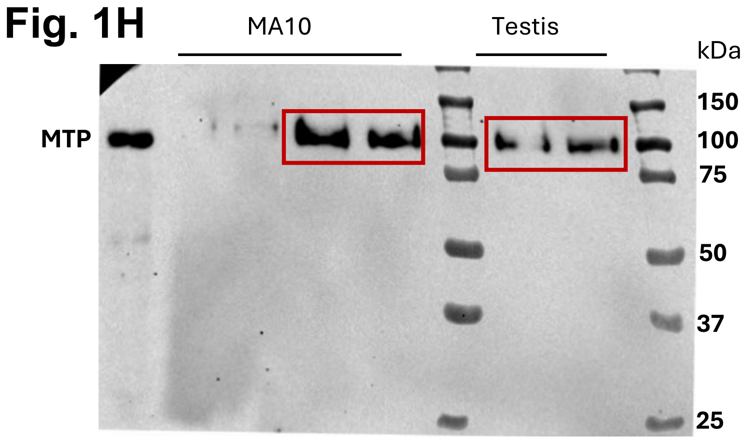

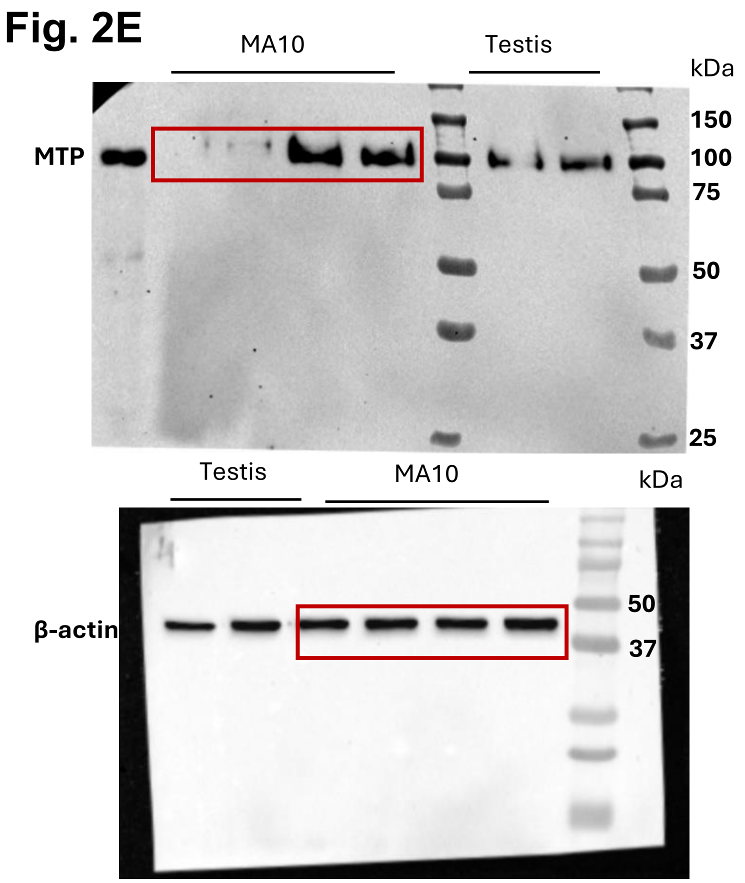

Fig. 3C

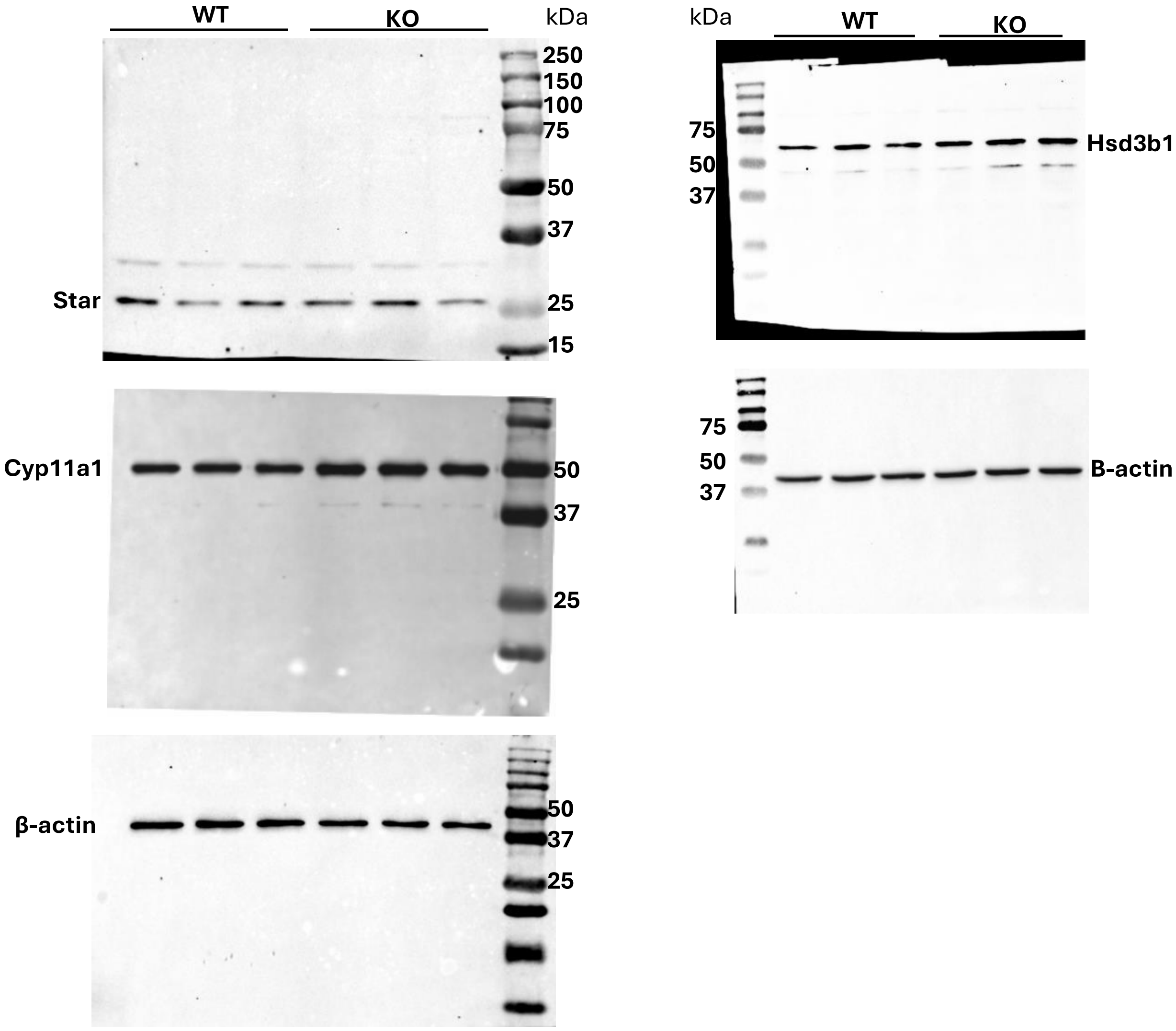

WT
KO
kDa
250
150
100
75
50
37
Star
25
15
Cyp11a1
50
37
25
β-actin
50
37
25
kDa
WT
KO
75
50
37
Hsd3b1
75
50
37
B-actin

Fig. 4F

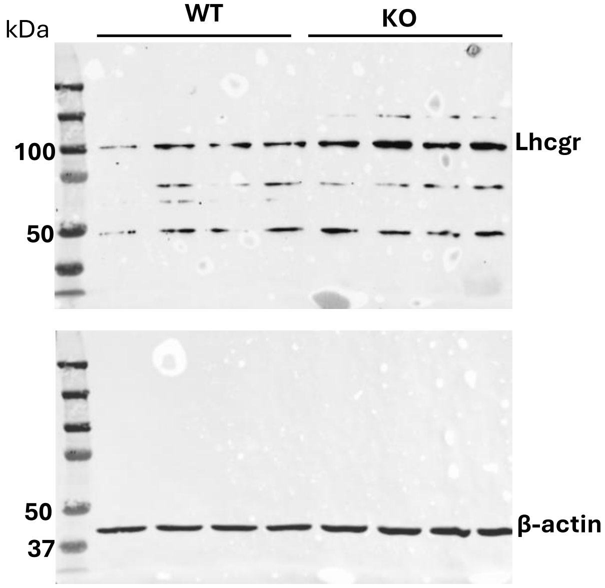

Fig. 4G

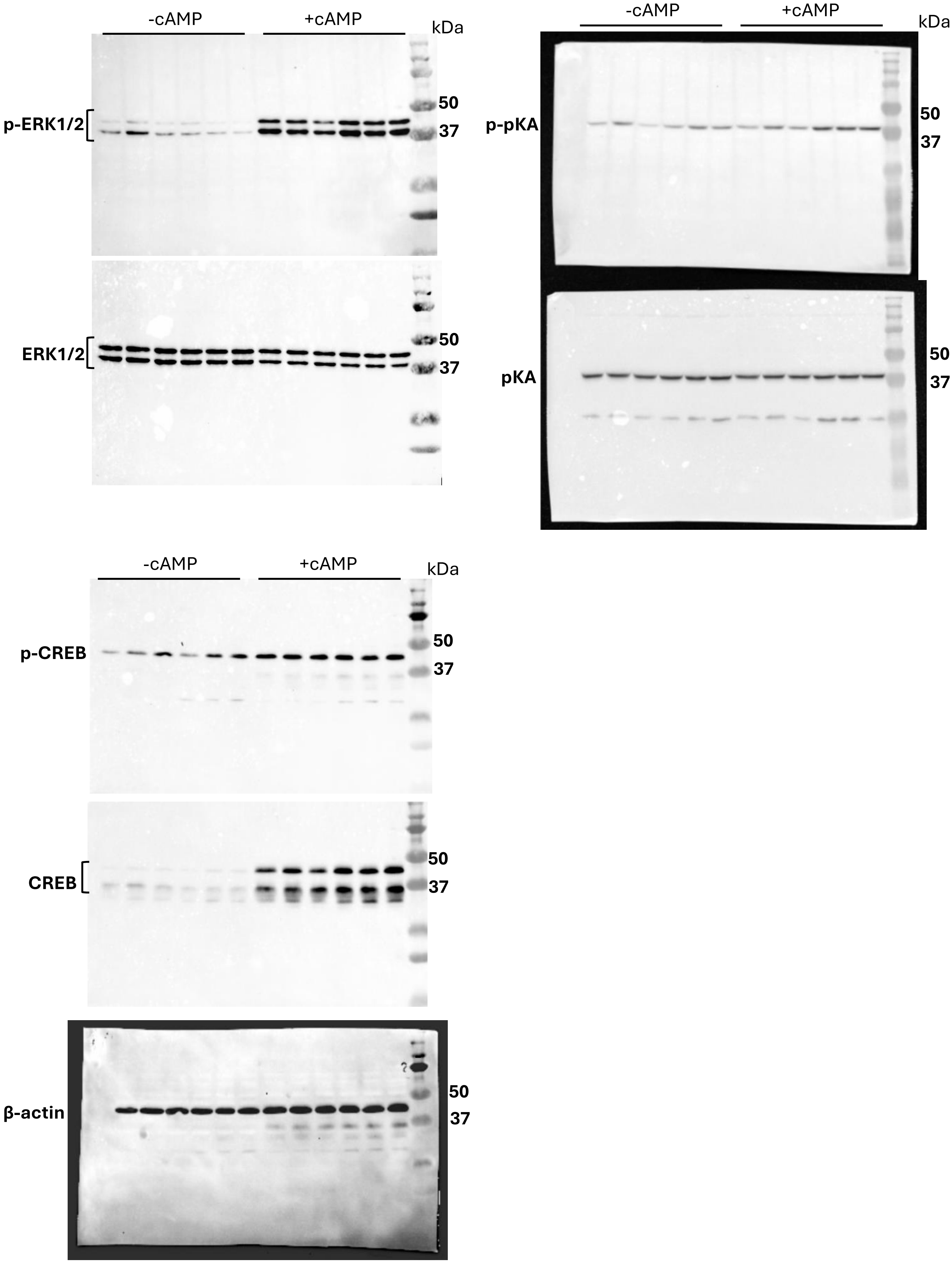

-cAMP
+cAMP
kDa
p-ERK1/2
50
37
ERK1/2
50
37
p-pKA
50
37
pKA
50
37
p-CREB
50
37
CREB
50
37
β-actin
50
37

Fig. 5B

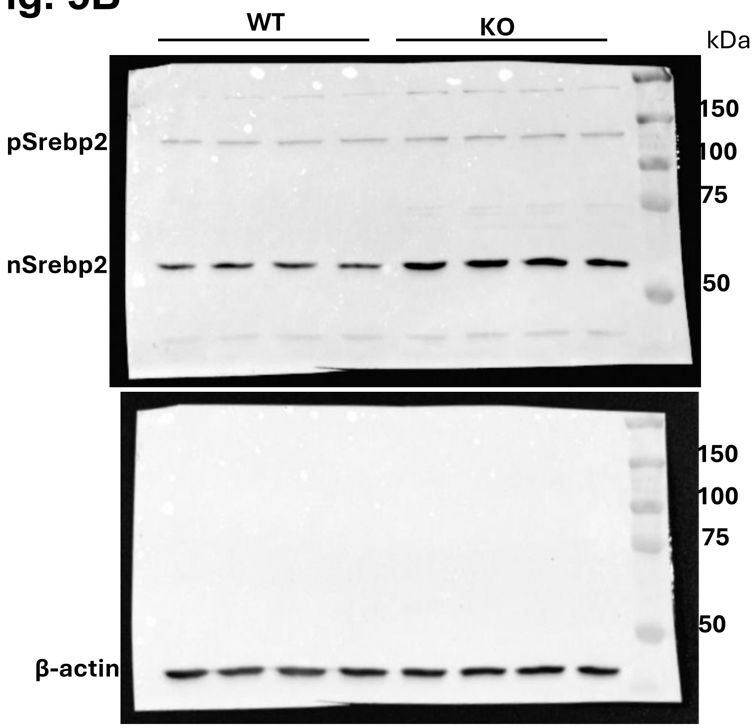

WT
KO
kDa
pSrebp2
nSrebp2
β-actin
150
100
75
50
150
100
75
50

Fig. 5C

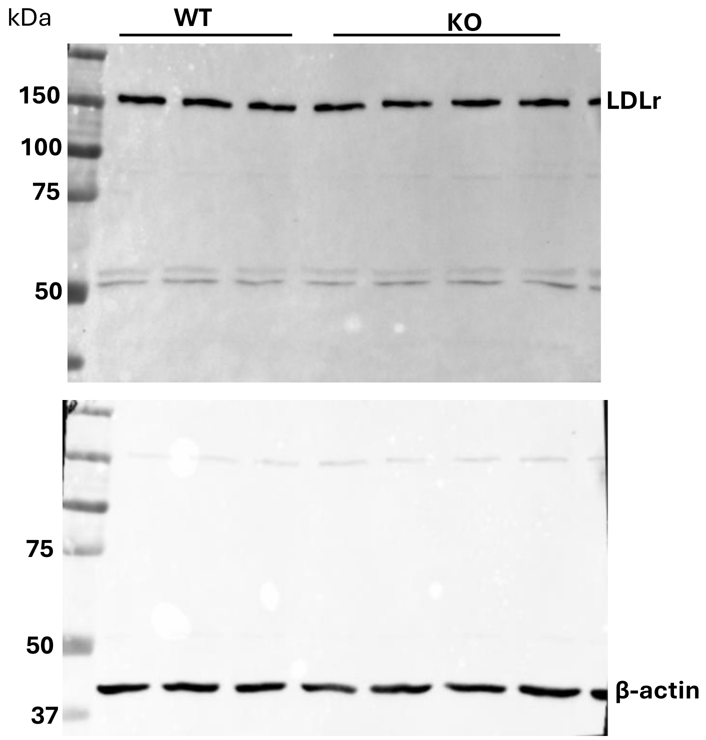

kDa
WT
KO
150
100
75
50
LDLr
75
50
37
β-actin
